# Cell-type-specific glutamine synthetase inhibition reveals the intercellular nitrogen economy in *Anabaena* sp. PCC 7120

**DOI:** 10.1093/plphys/kiag745

**Published:** 2026-10-06

**Authors:** David Malatinszky, Mattia Storti, Enrique Flores, Patrik Jones, Giorgio Perin

**Affiliations:** Department of Life Sciences, Imperial College London, South Kensington Campus, London SW7 2AZ, United Kingdom; Department of Biology, University of Padova, Via Ugo Bassi 58/B, Padova 35131, Italy; Instituto de Bioquímica Vegetal y Fotosíntesis, CSIC and University of Seville, Avda. Américo Vespucio 49, 41092 Seville, Spain; Department of Life Sciences, Imperial College London, South Kensington Campus, London SW7 2AZ, United Kingdom; Department of Life Sciences, Imperial College London, South Kensington Campus, London SW7 2AZ, United Kingdom; Department of Biology, University of Padova, Via Ugo Bassi 58/B, Padova 35131, Italy

## Abstract

Nitrogen homeostasis in heterocyst-forming filamentous diazotrophic cyanobacteria depends on the coordination between two cell types: heterocysts, which fix atmospheric N_2_, and vegetative cells, which carry out oxygenic photosynthesis. Yet the cell-type-specific contribution of glutamine synthetase (GS), the central enzyme of nitrogen assimilation, to this coordination remains poorly understood. We used cell-type-specific overexpression of IF7A, the GS posttranslational inhibitor, in both wild-type *Anabaena* sp. PCC 7120 and Δ*amt* genetic backgrounds, the latter lacking the high-affinity ammonium transporters (Amt) responsible for NH_4_^+^ uptake, to dissect the contribution of each cell type to the nitrogen economy of the filament. Whole-filament GS inhibition caused photosynthetic impairment, disruption of the NtcA–PII–PipX signaling axis, and growth arrest, establishing GS in vegetative cells as rate-limiting for filament growth. Heterocyst-targeted GS inhibition triggered accumulation of the nitrogen storage polymer cyanophycin, revealing that heterocyst GS governs the routing of fixed nitrogen either toward distribution or storage. Both phenotypes were amplified in the Δ*amt* background. Using pharmacological inhibition of GS with ammonium excretion measurements, we demonstrate that Amt transporters recover nitrogen lost by passive NH_3_ diffusion under diazotrophic conditions and that their absence renders the internal nitrogen economy chronically more vulnerable to GS inhibition. In natural nitrogen-limited environments, where diazotrophic cyanobacteria cannot compensate for passive nitrogen loss through external uptake, this recycling function is critical for nitrogen fixation efficiency. Overall, our findings redefine the architecture of intercellular nitrogen homeostasis in filamentous diazotrophic cyanobacteria, establishing GS and Amt transporters as interdependent determinants of intercellular nitrogen retention, distribution, and storage.

## Graphical Abstract

**Graphical Abstract:**
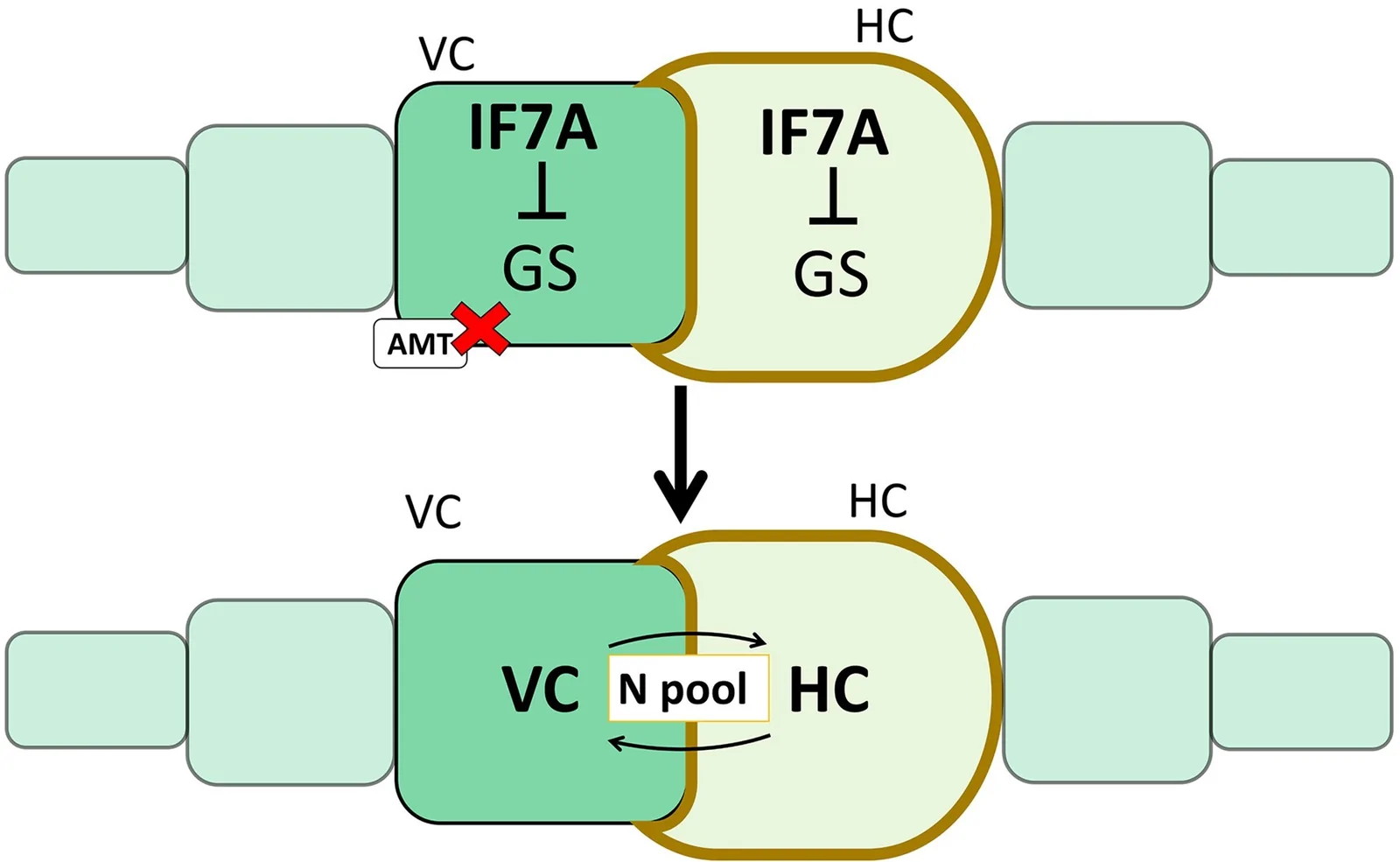

## Introduction

Diazotrophic cyanobacteria are ancient oxygenic phototrophs that have profoundly shaped the Earth's biogeochemical cycles and remain major contributors to global carbon (C) and nitrogen (N) fixation in contemporary ecosystems (Flores and Herrero 2010; Schirrmeister et al. 2013). Among them, the filamentous heterocyst-forming species *Anabaena* sp. PCC 7120 (hereafter *Anabaena*) represents a compelling model for studying cell differentiation, metabolic specialization, and intercellular communication in prokaryotes. Under combined nitrogen (N) deprivation, approximately every 10th cell along the filament irreversibly differentiates into a heterocyst, a terminally differentiated cell type that provides an anaerobic microenvironment for the oxygen-sensitive nitrogenase enzyme complex. This enables atmospheric N_2_ fixation to proceed concurrently with oxygenic photosynthesis in adjacent vegetative cells (Kumar et al. 2010; Flores et al. 2019b). This spatial division of metabolic labor is sustained by a continuous bidirectional exchange of metabolites between the two cell types: vegetative cells supply fixed C, primarily as sucrose, to heterocysts, which in return export fixed N, mostly in the form of glutamine (Gln), to the vegetative cells (Zhang et al. 2018; Magnuson 2019). Despite considerable progress in characterizing the molecular components of this exchange, the relative contributions of vegetative cells and heterocysts to the overall N economy of the filament remain incompletely resolved.

Central to N assimilation is the glutamine synthetase/glutamine oxoglutarate aminotransferase (GS/GOGAT) cycle, which converts ammonium (NH_4_^+^) into Gln and glutamate (Glu), which represent the primary organic N donors for biosynthetic pathways (Flores and Herrero 2005). Glutamine synthetase catalyzes the ATP-dependent condensation of NH_4_^+^ with Glu to form Gln and constitutes the main entry point for inorganic N into the metabolism. Given its central role, GS activity is subject to multilevel regulation, encompassing transcriptional, posttranscriptional, and posttranslational control mechanisms that together adjust the N assimilation capacity to the C–N balance of the cell (Forchhammer and Selim 2020). Among these, posttranslational inactivation of GS by small inhibitory factors, ie IF7 and IF17 in *Synechocystis* sp. PCC 6803 and IF7A in *Anabaena*, represents a rapid and reversible mechanism for dampening GS activity in response to N excess (García-Domínguez et al. 1999; Galmozzi et al. 2010).

The regulation of GS in cyanobacteria is embedded within a hierarchical signaling network with the metabolite 2-oxoglutarate (2-OG) at its apex, functioning as a molecular proxy of the intracellular C–N ratio (Zhang et al. 2018; Forchhammer and Selim 2020). When N is limiting, the concentration of 2-OG rises and its binding to the global transcription factor NtcA intensifies, enhancing its DNA-binding affinity and driving the transcriptional activation of genes required for N uptake, heterocyst differentiation, and N_2_ fixation (Vázquez-Bermúdez et al. 2002b; Picossi et al. 2004). Simultaneously, NtcA represses the expression of the gene *gifA*, encoding IF7A, thereby releasing GS from posttranslational inhibition when maximal N assimilation capacity is required (García-Domínguez et al. 1999; Flores et al. 2019b). The NtcA signaling output is further refined by the PII–PipX module: under N sufficiency, PII sequesters PipX, the coactivator of NtcA, dampening NtcA-dependent transcription; under N limitation, PII, upon binding with 2-OG, releases PipX, which associates with NtcA to stimulate its target genes (Espinosa et al. 2006; Llácer et al. 2008; Forchhammer and Selim 2020).

In *Anabaena*, the NtcA–PII–PipX signaling axis must be coordinated across two physiologically distinct cell types. As far as we are aware, the cell-type-specific contributions of GS activity to this signaling balance are not understood. The recent discovery of the nitroplast, a nitrogen-fixing organelle evolved from an endosymbiotic cyanobacterium in the marine alga *Braarudosphaera bigelowii* (Coale et al. 2024), has renewed interest in both understanding the molecular mechanisms that govern the efficiency of biological N_2_ fixation and the metabolic integration between N_2_-fixing compartments and their photosynthetic partners. The heterocyst–vegetative cell system of *Anabaena* is a well-characterized prokaryotic model for this type of inter-compartment N exchange, and understanding how GS activity governs the routing of fixed N within the filament has direct implications for understanding analogous processes in similar N_2_-fixing associations.

In this work, we constructed *Anabaena* strains overexpressing IF7A under the control of either the heterocyst-specific P*_nifHDK_* promoter or the whole-system P*_petE_* promoter, in both wild-type (WT) and Δ*amt* genetic backgrounds. The Δ*amt* mutant lacks the entire *amt* gene cluster responsible for ammonium uptake (Paz-Yepes et al. 2008) and displays a molecular fingerprint indicative of N deficiency even under N-replete conditions (Perin et al. 2021). Using a combination of growth and photosynthesis phenotyping, targeted proteomic quantification, enzyme activity assays, and pharmacological experiments with L-methionine sulfoximine (MSX), we demonstrate that heterocysts and vegetative cells have complementary roles in the N economy of the filament and provide experimental evidence for the role of Amt transporters in recycling N lost by passive diffusion out of the filament under diazotrophic conditions.

## Materials and methods

### Construction of IF7A overexpression *Anabaena* strains

The *asl2329* gene (also called *gifA*), encoding the posttranslational GS-inhibitory factor IF7A in *Anabaena*, was cloned into a pRSF1010-based broad-host–range shuttle vector derived from plasmid pVZ322 (kindly provided by Prof. Wolfgang Hess, University of Freiburg, Germany). The template pVZ322 plasmid contains two antibiotic resistance cassettes, *aphA* for kanamycin (Km^R^) and *aacC1* for gentamicin resistance (Gm^R^), which were both removed using primers RSF1010-bb-F (#1) and RSF1010-bb-R (#2) (Table S1) in a PCR linearization reaction, resulting in only the RSF1010 backbone. The vector backbone was combined with a Sm^R^ cassette conferring resistance to both streptomycin (St) and spectinomycin (Sp), amplified from plasmid pDF-lac (Guerrero et al. 2012) using primers Sm^R^-T*_gifA_*-F and Sm^R^-RSF1010-R (primers #3 and #4 in Table S1, respectively). The *asl2329* gene with its endogenous terminator and the plastocyanin (P*_petE_*) and nitrogenase (P*_nifHDK_*) promoters were amplified from high-purity genomic DNA of *Anabaena* sp. PCC 7120 using the primers listed in Table S1 (#5-#11).

DNA fragments consisting of the vector backbone, the Sm^R^ cassette, one of the two promoters, and the *asl2329* gene with its endogenous terminator were seamlessly assembled via Gibson cloning into chemically competent *E. coli* DH5α on St/Sp selective agar plates. In parallel, Gibson cloning was also exploited to place the coding sequence of YFP under the control of the two promoters to validate their cell-type specificity.

Isolated colonies were grown in 5 ml liquid LB medium supplemented with appropriate antibiotics, and plasmids were purified and sequenced (Source BioScience, Nottingham, UK) using primers RSF1010-Psch-seq-F (#12) or PSm^R^-seq-R (#13) (Table S1). Confirmed constructs were transformed into both *Anabaena* sp. PCC 7120 wild type and the Δ*amt* mutant by triparental conjugation. Exconjugants were isolated on BG-11 agar plates supplemented with 5 μg ml^−1^ St and Sp and verified by colony PCR using primers #12 to #13 (Table S1). Amplified fragments were gel-purified and sequenced for confirmation of the correct genotype.

### Cyanobacteria strains and growth conditions

*Anabaena* strains were maintained on solid BG11 medium (pH 8.0, supplemented with 10 mM TES–NaOH buffer [Rippka et al. 1979]) solidified with 1.5% Difco Bacto agar (BD) in an Algaetron 230 incubator (PSI, Photon Systems Instruments, Czech Republic) at 30 °C under cool white light at 60 μmol photons m^−2^ s^−1^ in an atmosphere containing 1% CO_2_. Prior to each experiment, strains were transferred to liquid BG11_0_ (BG11 lacking NaNO_3_) supplemented with 5 mM NH_4_Cl and grown axenically in 100-ml Erlenmeyer flasks at 160 rpm. Cultures were maintained in exponential phase by renewing half of the culture volume every second day. IF7A overexpression strains were grown with 5 μg ml^−1^ St and 5 μg ml^−1^ Sp. The Δ*amt* background additionally required 10 μg ml^−1^ neomycin. P*_petE_* strains were induced with 2 μM CuSO_4_. For all experiments, cells pre-grown in flasks were washed twice with BG11_0_ by centrifugation (10 min, 3,500 g, RT) and inoculated in 6-well polystyrene plates at OD_750_ = 0.1 in the same medium.

To pharmacologically inhibit GS activity, 3 µM L-Methionine-DL-sulfoximine (MSX) (Sigma-Aldrich) was added to the cultures at t = 0 h in 6-well plates under standard diazotrophic conditions, and cell-free supernatants were collected at each time point for ammonium quantification.

Growth was monitored by OD_750_ in 96-well plates using a Tecan Infinite M200 Pro spectrophotometer. A linear relationship between OD_750_ and biomass dry weight was verified for all strains and for all growth intervals examined in this work.

### Ammonium quantification

Ammonium/ammonia (NH_4_^+^/NH_3_) was quantified using a modified salicylate colorimetric method (WILLIS et al. 1996) adapted for 96-well microtiter plates. Cell-free supernatants (10 μl) were incubated with reactive solution (32 g L^−1^ sodium salicylate, 40 g L^−1^ Na_3_PO_4_·12H_2_O, 0.5 g L^−1^ sodium nitroprusside) and hypochlorite solution (0.25 to 0.37% active chlorine) for 15 min at 900 rpm, RT, and absorbance was read at 685 nm. Concentrations were determined from a NH_4_Cl standard curve. NH_4_^+^/NH_3_ excretion values were normalized over the nitrogenase activity.

### Confocal fluorescence microscopy

Cell-type-specific expression of the P*_nifHDK_* and P*_petE_* promoters was validated by confocal fluorescence microscopy using YFP reporter strains. Filaments grown under diazotrophic conditions were mounted on glass slides and imaged using a Zeiss LSM 900 confocal laser scanning microscope, available at the Imaging Facility of the Department of Biology, University of Padova. YFP fluorescence was excited at 405 nm and detected in the 501 to 537 nm range. Chlorophyll (Chl) was excited at 488 nm and fluorescence detected in the 656 to 700 nm range. Bright field images were acquired simultaneously. All images were processed using Zeiss ZEN software.

### Pigment content and photosynthetic efficiency

Pigments were extracted from intact cells using 100% methanol (1:1 biomass–solvent ratio) at 4 °C for ≥20 min (Sinetova et al. 2012). Absorbance at 470, 665, and 730 nm was recorded to quantify Chl and carotenoid (Car) content using specific extinction coefficients (Wellburn 1994; Ritchie 2006). Apparent photosystem II maximum quantum yield (apparent Φ_PSII_) was measured on intact cells using an AquaPen-C AP 110-C (PSI, Photon Systems Instruments, Czech Republic), with a 630 nm excitation LED, following (Maxwell and Johnson 2000). Values are reported as apparent Φ_PSII_, because the high proportion of non-PSII fluorescence in cyanobacteria, including contributions from phycobilisomes and shared photosynthetic–respiratory electron transport pathways, precludes accurate determination of absolute PSII quantum yield by this method (Wey et al. 2026).

### Glutamine synthetase activity assay

Glutamine synthetase activity was measured by the biosynthetic assay (Orr et al. 1981; Bressler and Ahmed 1984; Mérida et al. 1991). Cell pellets were disrupted twice in a TissueLyser II (Qiagen) with acid-washed glass beads in solubilization buffer (50 mM Hepes, 0.2 mM EDTA, pH 7.3) for 5 min at 30 Hz with prechilled holders (−20 °C). Clarified extracts (2× centrifugation at 21,000 g, 4 °C) were assayed at 50 μg total protein in 364 mM imidazole–HCl pH 7.0, 1.82 mM NH_4_Cl, 5.45 mM Na-ATP·H_2_O pH 7.0, 0.52 M MgCl_2_·6H_2_O, and 91 mM sodium glutamate pH 7.0 for 15 min at 30 °C. Phosphate released was quantified colorimetrically at OD_850_ after addition of FeSO_4_ and ammonium heptamolybdate. Activity is expressed as ΔOD_850_ corrected against a glutamate-free blank.

### Nitrogenase activity assay

Nitrogenase activity was measured by the acetylene reduction assay. Cells were transferred to 2-ml GC vials under 10% acetylene in air and incubated for 3 h under standard growth conditions. Ethylene in the headspace was quantified by gas chromatography (7820A GC system, Agilent Technologies). Activity is expressed as % ethylene/acetylene h^−1^ μg Chl^−1^.

### Cyanophycin quantification

Cyanophycin (CGP) content was determined as previously described (Perin et al. 2021), with minor modifications. Briefly, the same amount of biomass (OD_750_ = 0.3) was harvested by centrifugation, and the cell pellet was resuspended in 1 ml 100% acetone and incubated for 30 min at 1,400 rpm, RT. After centrifugation (17 min, 21,000g, RT), the pellet was resuspended in 1.5 ml 0.1 M HCl and incubated for 1 h at 1,400 rpm, 60 °C, to solubilize CGP polymers. Insoluble material was removed by centrifugation (17 min, 21,000g, RT), Tris–HCl pH 8.0 was added to the supernatant (0.2 M final concentration), and samples were incubated for 40 min at 4 °C. Pelleted CGP polymers were recovered by centrifugation (17 min, 21,000g, 4 °C) and resuspended in 500 µl 0.01 M HCl for quantification. Since CGP is a polymer of arginine and aspartate (Watzer and Forchhammer 2018), its content was determined by quantifying the arginine released from the polymer using a modified colorimetric Sakaguchi method (Messineo 1966), as previously used in *Anabaena* (Burnat et al. 2014; Perin et al. 2021).

### Targeted proteomic analysis

Cell pellets were lysed in extraction buffer (20 mM Tris–HCl pH 8.0, 1 mM EDTA, 2 mM DTT) using a TissueLyser II with glass beads (2 × 5 min at 30 Hz, −20 °C holders). Clarified extracts were diluted to 0.3 μg μl^−1^ total protein in 0.1 M ammonium bicarbonate containing 0.3 ng μl^−1^ [Glu1]-Fibrinopeptide B (GluFib, Sigma-Aldrich) as internal standard. Samples were reduced (10 mM DTT, 56 °C, 1 h), alkylated (50 mM iodoacetamide, 37 °C, 30 min), and digested overnight with trypsin (6 ng μl^−1^, 37 °C). Digestion was quenched with 1% formic acid and insoluble material removed by centrifugation.

Tryptic peptides were analyzed by MRM on an AB-SCIEX 6500 QTrap coupled to an Agilent 1100 LC system using a Phenomenex Luna C18(2) column (100 × 2 mm, 3 μm) at 50 °C, following (Perin et al. 2021). Gradient: 0 to 35% B (94.9% CH_3_CN/5% H_2_O/0.1% formic acid) over 30 min at 250 μl min^−1^. Protein quantification used summed MRM peak areas for all peptides per protein, normalized to GluFib intensity. Signature peptides are listed in Table S2.

### Statistical analysis

Statistical significance was evaluated using Student's *t*-test in OriginPro 2025b (v. 10.2.5.212). Sample size was ≥4 for all measurements.

## Results

### Construction and validation of cell-type-specific IF7A overexpression strains

To investigate the cell-type-specific roles of GS in *Anabaena*, we first validated the expression specificity of the two selected promoters by confocal fluorescence microscopy with YFP reporter strains grown under diazotrophic conditions. In the P*_petE_*::YFP strain, YFP fluorescence was observed uniformly across all cells of the filament, including both vegetative cells and heterocysts. In the P*_nifHDK_*::YFP strain, YFP fluorescence was restricted exclusively to heterocysts, with no detectable signal in adjacent vegetative cells (Fig. 1).

**Figure 1:**
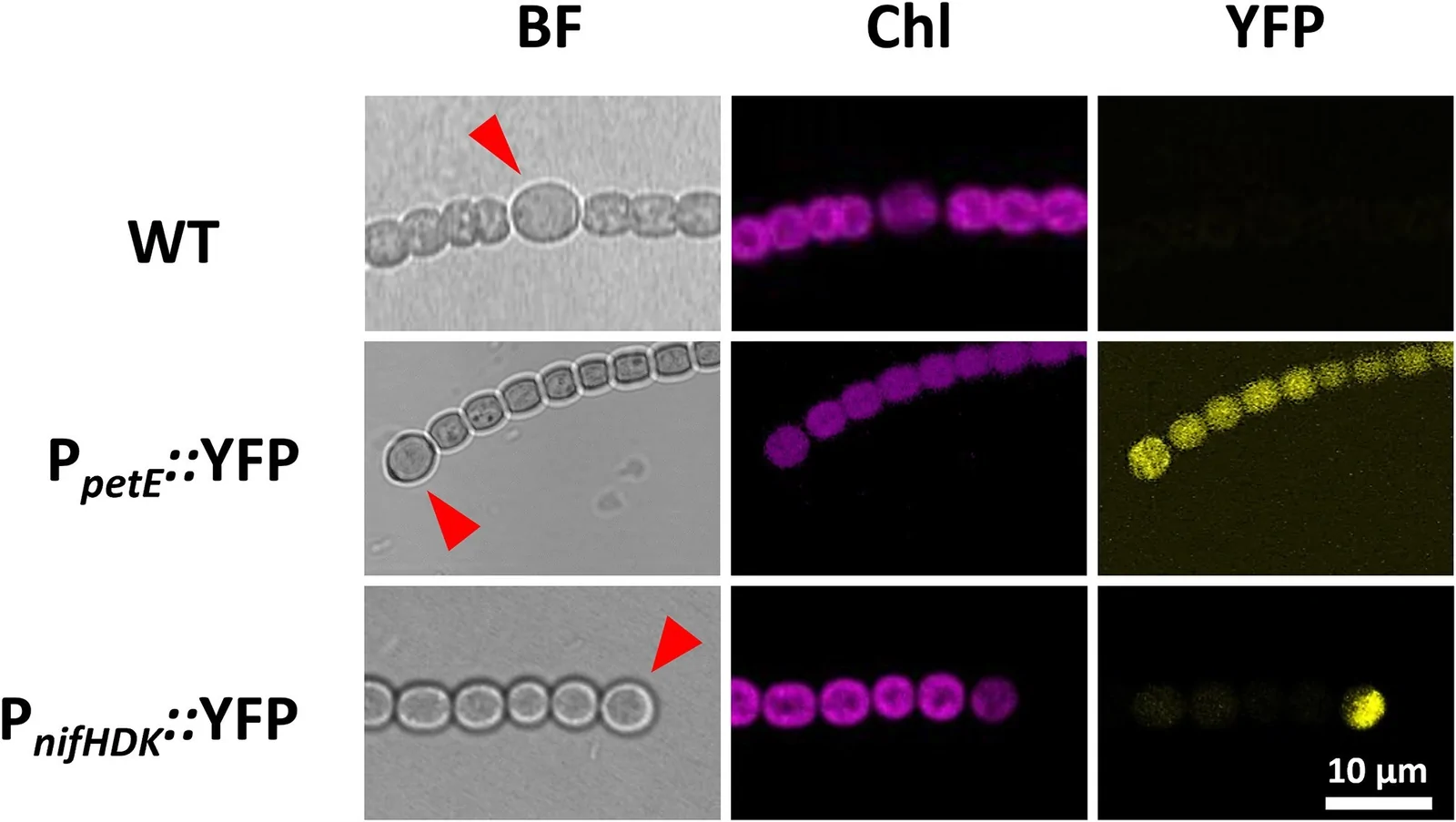
Validation of P*_nifHDK_* and P*_petE_* promoter cell-type-specific expression. Filaments of wild-type (WT), P*_petE_*::YFP, and P*_nifHDK_*::YFP *Anabaena* reporter strains were imaged under diazotrophic growth conditions. For each strain, three channels are shown: bright field (BF), chlorophyll autofluorescence (Chl, magenta), and YFP fluorescence (YFP, yellow). Red arrowheads indicate heterocysts, identifiable in the BF channel by their enlarged, round morphology and thickened cell envelope. Scale bar = 10 µm.

The endogenous *asl2329* gene (*gifA*), encoding IF7A, was then episomally expressed under the control of either P*_nifHDK_* or P*_petE_* in both WT and Δ*amt* backgrounds. Successful transformation was verified by colony PCR (Figure S1).

### IF7A overexpression differentially affects diazotrophic growth and photosynthesis in a cell-type-specific manner

The abundance of IF7A was quantified by targeted proteomics in both the WT and Δ*amt* genetic backgrounds. It was approximately 2-/3-fold higher in both the P*_nifHDK_* and P*_petE_* overexpression lines relative to their respective parental strains (Fig. 2a and b). Notably, basal IF7A levels were significantly lower in the Δ*amt* genetic background than in the WT (Fig. 2b), consistent with the previously described N-limited internal metabolic state of this mutant (Perin et al. 2021). It should be noted that while P*_nifHDK_*::*gifA* drives IF7A overexpression exclusively in heterocysts and P*_petE_*::*gifA* in both cell types, all physiological and proteomic measurements reported hereafter were performed on whole-filament extracts and therefore reflect the integrated response of the entire biological system.

**Figure 2:**
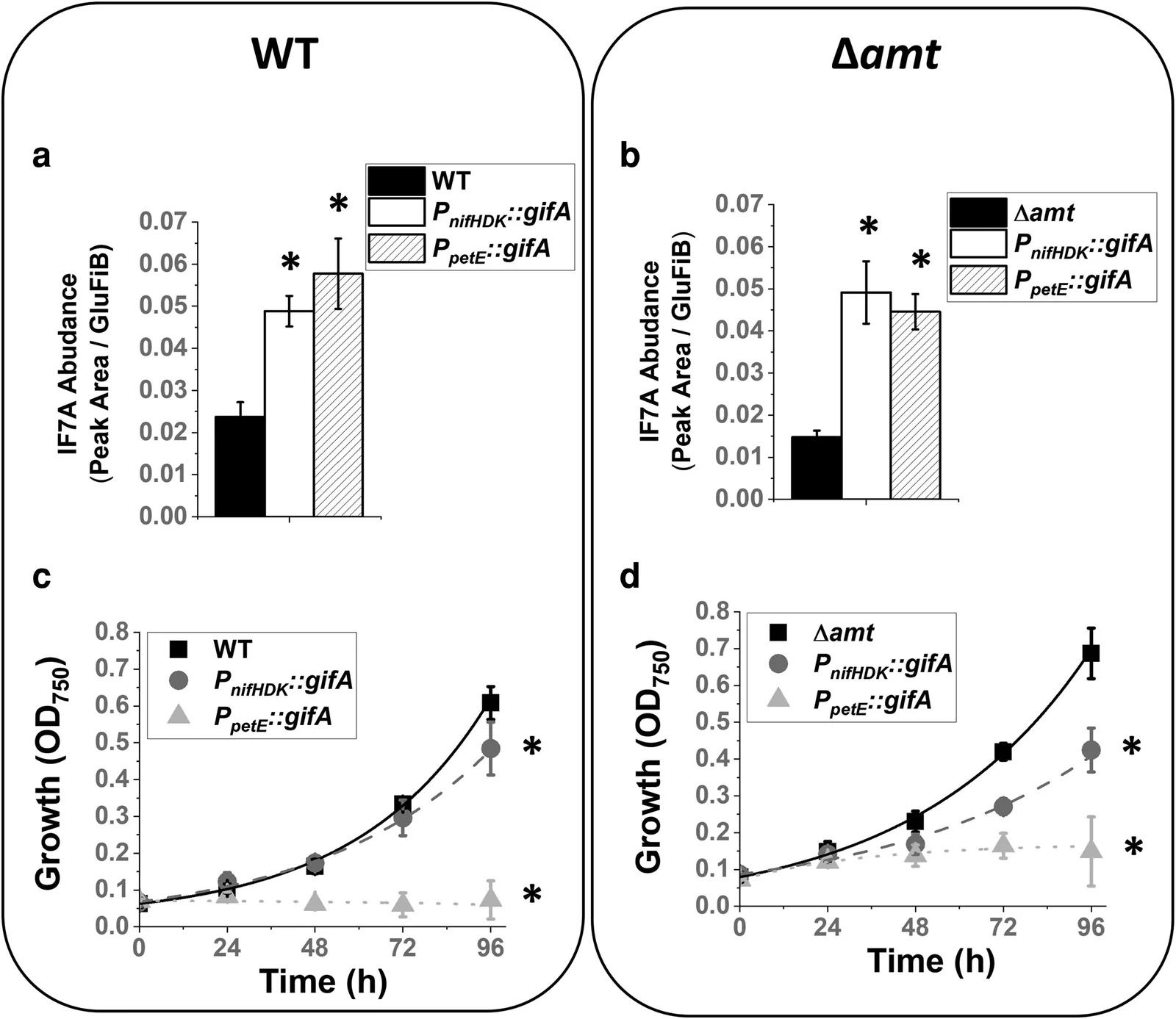
IF7A content and growth of *Anabaena* strains selectively overexpressing IF7A. (a, b) Abundance of IF7A, normalized over the GluFiB internal standard, in *Anabaena* strains carrying either a heterocyst-specific (P*_nifHDK_::gifA*, white bar) or whole-filament (P*_petE_::gifA*, dashed lines bar) overexpression of IF7A in the WT (a) and Δ*amt* genetic background (b) after 96 h of growth in diazotrophic conditions. Growth, expressed as optical density at 750 nm (OD_750_), over a 96-h time window upon diazotrophic conditions for all the strains of this work either in the WT (c) or Δ*amt* genetic background (d). Asterisks indicate statistically significant differences between IF7A overexpressing lines and the respective parental strain within the same genetic background (Student's t-test, *P*-value < 0.05).

Under diazotrophic growth conditions, the strain overexpressing IF7A under the control of the heterocyst-specific promotor (P*_nifHDK_*::*gifA*) in the WT background showed a moderate but significant growth reduction relative to WT (Fig. 2c). By contrast, the strain overexpressing IF7A in the whole filament (P*_petE_*::*gifA*) displayed near-complete growth arrest, remaining below OD_750_ ≈0.1 throughout 96 h. In the Δ*amt* genetic background, P*_nifHDK_*::*gifA* showed a more pronounced growth reduction than the WT (OD_750_ ≈0.4 versus 0.7 in Δ*amt* at 96 h; Fig. 2d), while P*_petE_*::*gifA* again caused near-complete growth arrest, as observed in the WT genetic background. The consistently more severe growth impairment when GS activity was inhibited in vegetative cells suggests that it is rate-limiting for growth of the diazotrophic filament.

The selective inhibition of N assimilation also affected the photosynthetic properties of the filaments. The heterocyst-specific inhibition of GS in the WT background caused a moderate, yet significant, increase in both Chl and Car content without affecting the Chl/Car ratio (Table 1), consistent with a photosynthetic compensatory response to reduced N assimilation in heterocysts. In contrast, the whole-filament inhibition of GS caused 50% reductions in both Chl and Car content accompanied by a significant reduction of the Chl/Car ratio (Table 1), suggesting photodamage consistent with N starvation in vegetative cells. In the Δ*amt* genetic background, the heterocyst-specific GS inhibition resulted in 50% reduction of the Chl/Car ratio through a significant decrease in Chl content, while the whole-filament GS inhibition caused >75% reduction of the Chl/Car ratio, because of a severe reduction in both the Chl and Car contents (Table 1). Consistently, these modifications in the composition of pigments affected also the apparent maximum quantum yield of PSII (apparent Φ_PSII_). Apparent Φ_PSII_ was significantly reduced only by the whole-filament GS inhibition in the WT background, whereas both overexpression lines reduced the apparent Φ_PSII_ significantly in the Δ*amt* background (Table 1).

**Table 1:** Pigment content and apparent PSII maximum quantum yield (apparent Φ_PSII_).

	WT	Δ*amt*
**Chl content (µg/OD_750_)**		
Parental strain	12.06 ± 0.82	13.44 ± 0.68
P*_nifHDK_:: gifA*	15.35 ± 0.49^a^	7.74 ± 0.25^a^
P*_petE_:: gifA*	4.72 ± 0.67^a^	2.51 ± 0.19^a^
**Car content (µg/OD_750_)**		
Parental strain	4.78 ± 0.58	4.71 ± 0.5
P*_nifHDK_:: gifA*	6.37 ± 0.21^a^	5.4 ± 0.14
P*_petE_:: gifA*	2.59 ± 0.52^a^	3.62 ± 0.02^a^
**Chl/Car**		
Parental strain	2.59 ± 0.13	2.83 ± 0.27
P*_nifHDK_:: gifA*	2.41 ± 0.02	1.43 ± 0.009^a^
P*_petE_:: gifA*	1.84 ± 0.12^a^	0.69 ± 0.05^a^
**Apparent Φ_PSII_**		
Parental strain	0.67 ± 0.02	0.67 ± 0.03
P*_nifHDK_:: gifA*	0.67 ± 0.005	0.5 ± 0.002^a^
P*_petE_:: gifA*	0.5 ± 0.01^a^	0.49 ± 0.001^a^

Data were collected after 96 h of cultivation under diazotrophic conditions and are expressed as mean ± SD of at least 3 biological replicates. ^a^Statistically significant differences between IF7A overexpressing lines and the respective parental strain within the same genetic background (Student's t-test, *P*-value < 0.05).

### The selective overexpression of IF7A differentially impacts GS and nitrogenase activity

Whole-filament GS activity, measured by the biosynthetic assay (see Methods for details), was not significantly affected by IF7A overexpression in the WT background, regardless of the cell specificity of the inhibition (Fig. 3a).

**Figure 3:**
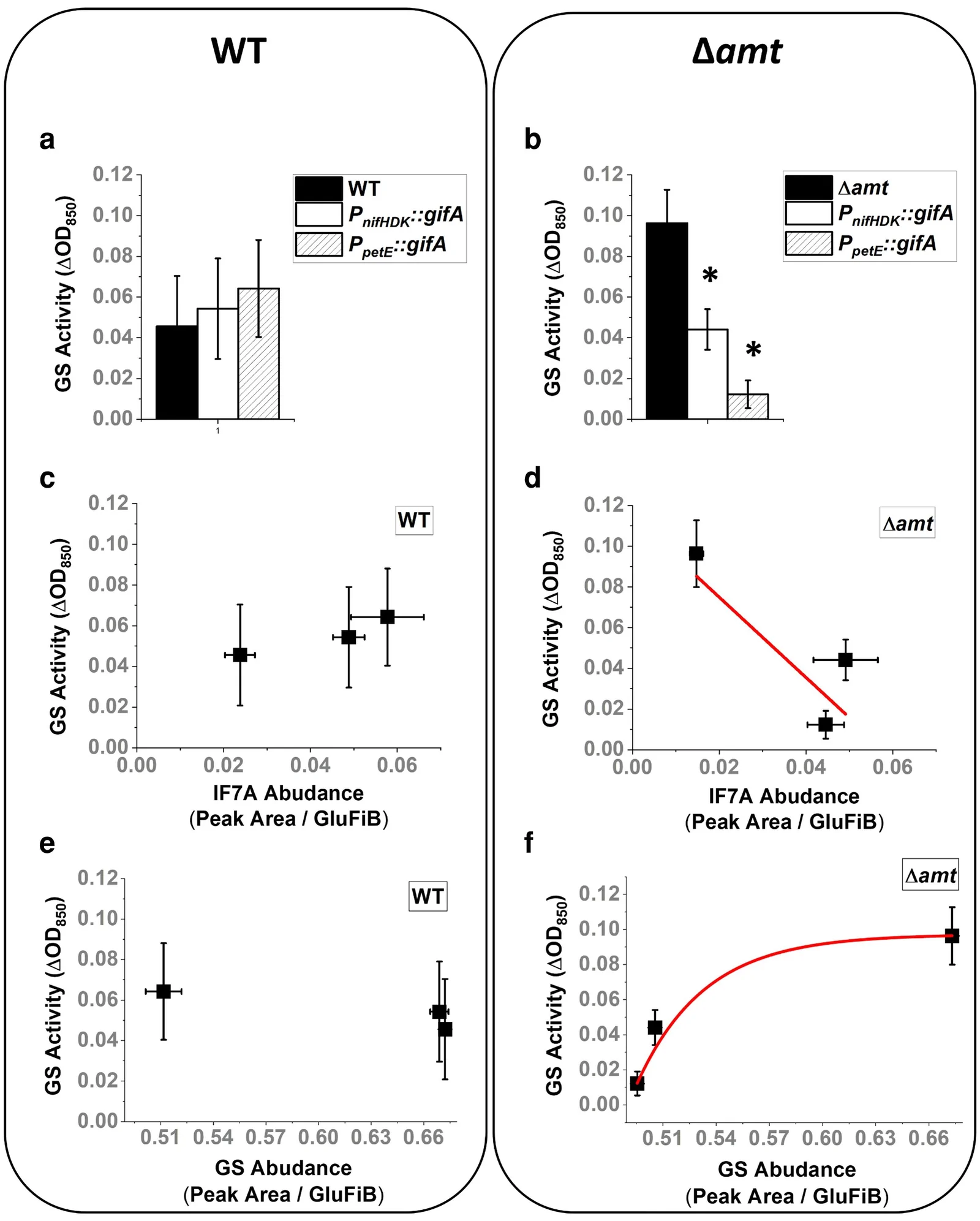
Impact of IF7A overexpression on GS activity. Whole-filament glutamine synthetase (GS) activity (a, b) and correlation with the IF7A protein abundance (c, d) in wild-type (WT, a, c) and Δ*amt* (b, d) genetic backgrounds. (a, b) GS activity measured by the biosynthetic assay (expressed as ΔOD_850_) in WT (a) and Δ*amt* (b) parental strains and the corresponding P*_nifHDK_::gifA* (white bars) and P*_petE_::gifA* (dashed lines bar) overexpression lines, after 96 h of growth in diazotrophic conditions. (c, d) Scatter plots showing GS activity as a function of IF7A protein abundance (peak area/GluFiB) for all three strains in the WT (c) and Δamt (d) backgrounds. Each data point represents a strain. No significant correlation is observed in the WT background (c). A significant negative linear correlation between IF7A abundance and GS activity is observed exclusively in the Δ*amt* background (d, red regression line). (e, f) Correlation between GS activity (ΔOD_850_) and GS protein abundance (peak area/GluFiB) in WT (e) and Δ*amt* (f) backgrounds. The red curve in panel F represents a saturation kinetics fit. Each data point represents one strain. Data are expressed as mean ± SD of at least three biological replicates. IF7A abundances is normalized to the internal standard [Glu1]-Fibrinopeptide B (GluFiB). Asterisks indicate statistically significant differences between IF7A overexpressing lines and the respective parental strain within the same genetic background (Student's t-test, *P*-value < 0.05).

On the contrary, in the Δ*amt* background, GS activity was significantly reduced in both overexpression lines, and the heterocyst-specific inhibition caused a 50% reduction, while the whole-filament inhibition caused a 90% reduction relative to the parental Δ*amt* strain (Fig. 3b). Plotting GS activity against IF7A abundance revealed a clear negative linear correlation exclusively in the Δ*amt* background (Fig. 3d), with no significant correlation detectable in the WT (Fig. 3c), suggesting that the inhibitory effect of IF7A on GS activity is likely masked either by the presence of Amt transporters or by their regulatory role in the WT strain.

At the protein level, the heterocyst-specific inhibition did not affect the whole-filament GS abundance in the WT background, whereas the whole-filament inhibition caused a 25% reduction (Figure S2a). In the Δ*amt* background, GS abundance was reduced by 25% in both overexpression lines (Figure S2b), suggesting that IF7A overexpression in vegetative cells triggers also secondary proteolytic degradation of GS, consistent with the protein turnover upon sustained inactivation reported in *Synechocystis* (Galmozzi et al. 2010). Therefore, to distinguish whether IF7A overexpression triggers a decrease either in the protein abundance or in its specific activity, we plotted GS activity against GS protein abundance (Fig. 3e and f). In the WT background, no correlation was observed (Fig. 3e), indicating that IF7A acts primarily by inhibiting the specific activity of the enzyme, consistent with the known mechanism of direct protein–protein interaction (García-Domínguez et al. 1999; Galmozzi et al. 2010). In the Δ*amt* background, by contrast, a positive correlation, with a saturation kinetics profile, was observed (Fig. 3f), reflecting a combined effect of reduced enzyme quantity and IF7A-mediated inhibition of GS specific activity operating simultaneously. Interestingly, these two mechanisms become coupled below a critical threshold of GS abundance that is reached in the Δ*amt* overexpression strains but not in the WT.

GOGAT abundance, which is only present in vegetative cells, showed instead a divergent pattern, and in the WT background, the heterocyst-specific inhibition increased GOGAT by ∼45%, while the whole-filament inhibition had no effect (Figure S2c). In the Δ*amt* background, GOGAT was strongly reduced in both overexpression lines, with a > 50% reduction upon heterocyst-specific inhibition (Figure S2d). This reduction of GOGAT in vegetative cells when GS is inhibited in heterocysts in the Δ*amt* background establishes a direct coupling between heterocyst GS activity and the downstream N assimilation machinery of the whole filament.

Nitrogenase activity, which is instead exclusive to heterocysts, measured by the acetylene reduction assay (see Methods for details), was significantly reduced in both overexpression lines and both genetic backgrounds (Fig. 4a and b).

**Figure 4:**
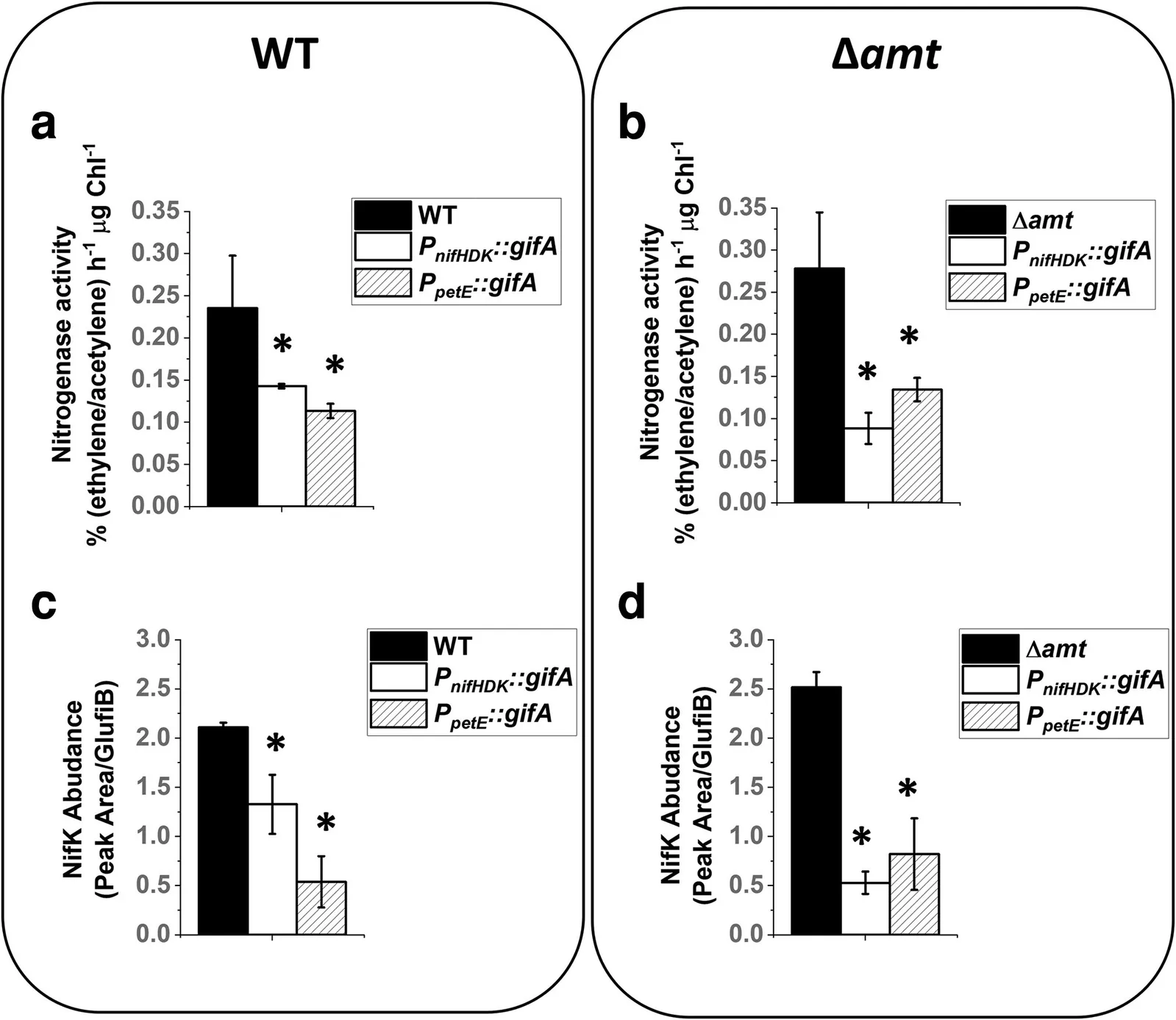
Nitrogenase activity and abundance. (a, b) Nitrogenase activity measured by the acetylene reduction assay (ARA), expressed as % ethylene/acetylene h^−1^ µg Chl^−1^, in WT (a) and Δ*amt* (b) parental strains (black bars) and the corresponding P*_nifHDK_::gifA* (white bars) and P*_petE_::gifA* (dashed lines bars). (c, d) Abundance of the nitrogenase β-subunit NifK (peak area/GluFiB) in WT (c) and Δ*amt* (d) parental strains and the corresponding P*_nifHDK_::gifA* and P*_petE_::gifA* overexpression lines. Data were collected after 96 h of growth in diazotrophic conditions and are expressed as mean ± SD of at least three biological replicates. Protein abundances are normalized to the internal standard GluFiB. Asterisks indicate statistically significant differences between IF7A overexpressing lines and the respective parental strain within the same genetic background (Student's t-test, *P*-value < 0.05).

In both genetic backgrounds, both P*_nifHDK_*::*gifA* and P*_petE_*::*gifA* showed >40% reduction of the nitrogenase activity with respect to the parental strains (Fig. 4a and b), suggesting that GS plays a role in heterocysts that influences the rate of N_2_ fixation. The accumulation of the nitrogenase structural subunits NifK (Fig. 4c and d) and NifD (Figure S3a and b) was also reduced, suggesting a downregulation of the nitrogenase complex itself.

### IF7A overexpression perturbs the NtcA–PII–PipX signaling axis

N_2_ fixation and assimilation activities in *Anabaena* are controlled by a sophisticated signaling axis, shared by both cell types, based on the interaction between the NtcA, PII, and PipX proteins, which is coordinated by the C–N balance of the cell (Forchhammer and Selim 2020). In the WT background, the heterocyst-specific GS inhibition did not affect NtcA or PII abundance (Fig. 5a and c) but reduced PipX by ∼25% (Fig. 5e) in the whole filament.

**Figure 5:**
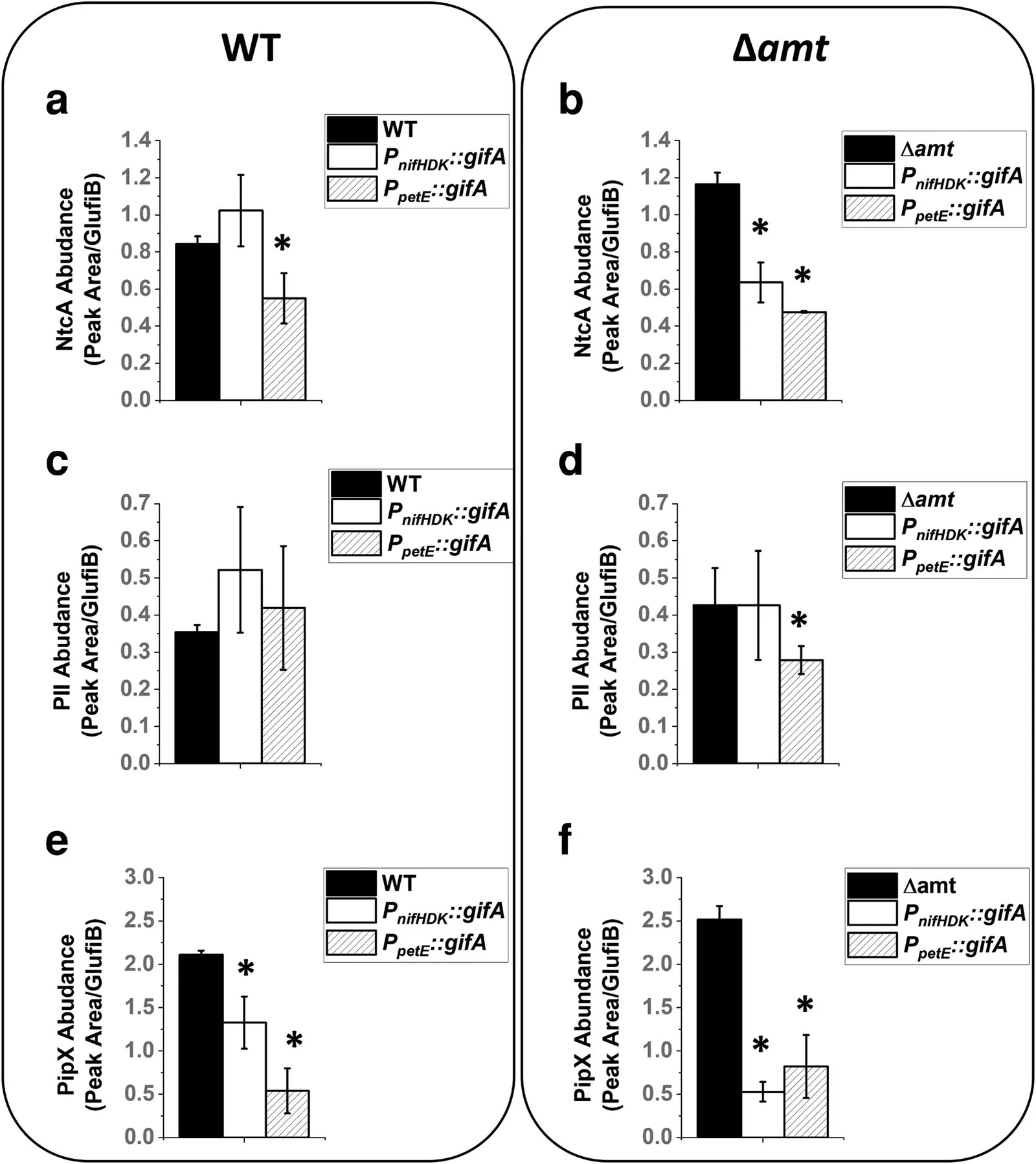
Abundance of the NtcA–PII–PipX C/N signaling axis. (a, b) NtcA abundance in WT (a) and Δ*amt* (b) parental strains (black bars) and the corresponding P*_nifHDK_::gifA* (white bars) and P*_petE:_:gifA* (dashed lines) overexpression lines. (c, d) PII abundance in WT (c) and Δ*amt* (d) parental strains and the corresponding P*_nifHDK_::gifA* and P*_petE:_:gifA* overexpression lines. (e, f) PipX abundance in WT (e) and Δ*amt* (f) parental strains and the corresponding P*_nifHDK_::gifA* and P*_petE:_:gifA* overexpression lines. Data were collected after 96 h of growth in diazotrophic conditions and are expressed as mean ± SD of at least three biological replicates. Protein abundances are normalized to the internal standard GluFiB. Asterisks indicate statistically significant differences between IF7A overexpressing lines and the respective parental strain within the same genetic background (Student's t-test, *P*-value < 0.05).

On the other hand, the whole-filament GS inhibition caused more severe effects, reducing PipX by ∼75% (Fig. 5e) and significantly reducing NtcA abundance, as well (Fig. 5a). In the Δ*amt* background, the heterocyst-specific inhibition caused 50% and 80% reductions in NtcA and PipX, respectively (Fig. 5b and f). The whole-filament GS inhibition intensified all three effects, also causing a significant reduction of PII in the Δ*amt* background (Fig. 5d). Overall, PipX was consistently reduced across all four overexpression lines, regardless of the promoter or the genetic background. It is worth noting that PipX is required as a coactivator of NtcA; therefore, its reduction is expected to dampen the full NtcA-dependent gene activation response. This provides a direct mechanistic link between posttranslational GS inhibition and attenuation of NtcA-dependent transcriptional responses, including N_2_ fixation and assimilation genes.

### IF7A overexpression redirects fixed nitrogen toward cyanophycin storage

Cyanophycin (CGP) is a nitrogen-rich polymer of arginine and aspartate that accumulates at the heterocyst poles in filamentous cyanobacteria (Sherman et al. 2000; Nieves-Morión et al. 2021). It acts as a transient buffer for fixed N before its export to vegetative cells as Gln (Burnat et al. 2014; Watzer and Forchhammer 2018). Although CGP represents a minor N sink relative to the bulk protein pool (Simon 1973), its strategic localization at the intercellular junction positions it as a regulator of the N flux between heterocysts and vegetative cells.

In the WT background, the CGP content of the whole filament was approximately 2-fold higher in both P*_nifHDK_::gifA* and P*_petE_::gifA* relative to the WT parental strain (Fig. 6a). The effect was even more pronounced in the Δ*amt* genetic background, where both overexpression lines accumulated approximately 10-fold more CGP relative to the Δ*amt* parental strain (Fig. 6b).

**Figure 6:**
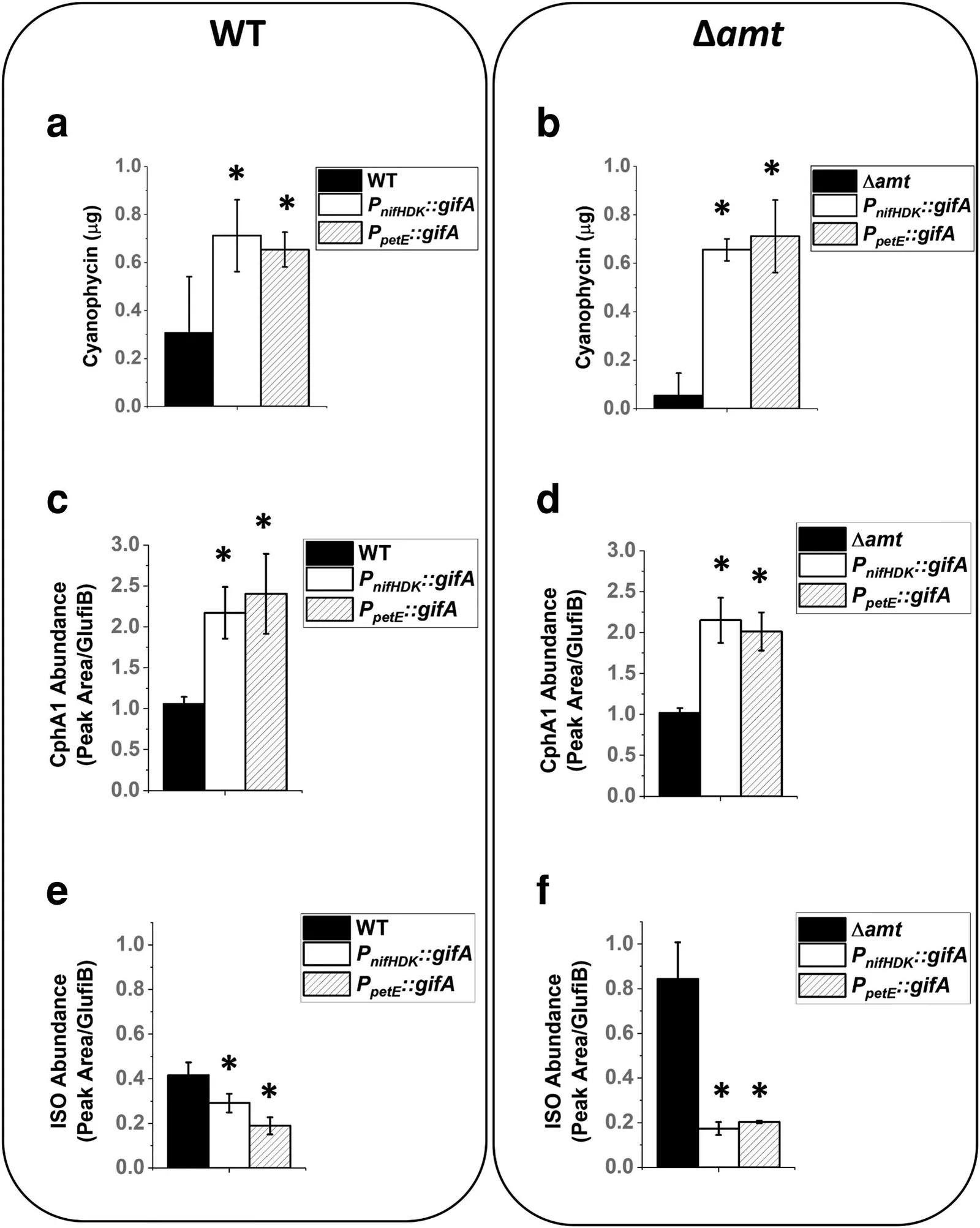
Cyanophycin content and abundance of the enzymes of its metabolism. Cyanophycin content in WT (a) and Δ*amt* (b) parental strains (black bars) and the corresponding P*_nifHDK_::gifA* (white bars) and P*_petE_::gifA* (dashed line bars) overexpression lines after 96 h of growth in diazotrophic conditions. The same amount of biomass was processed for all strain (see Methods for details). Abundance of cyanophycin synthetase 1 (CphA1) and isoaspartyl dipeptidase (ISO) in all the strains investigated in this work. (c, d) CphA1 abundance in WT (c) and Δ*amt* (d) parental strains (black bars) and the corresponding P*_nifHDK_::gifA* (white bars) and P*_petE_::gifA* (dashed line bars) overexpression lines. (e, f) ISO abundance in WT (e) and Δ*amt* (f) parental strains and the corresponding P*_nifHDK_::gifA* and P*_petE_::gifA* overexpression lines. Data were collected after 96 h of growth in diazotrophic conditions and are expressed as mean ± SD of at least three biological replicates. Protein abundances are normalized to the internal standard GluFiB. Asterisks indicate statistically significant differences between IF7A overexpressing lines and the respective parental strain within the same genetic background (Student's t-test, *P*-value < 0.05).

The CGP accumulation trend was paralleled by the upregulation of CphA1, the principal heterocyst-localized CGP synthetase, which increased more than 2-fold in both overexpression lines relative to their respective parental strains, in both genetic backgrounds (Fig. 6c and d).

In marked contrast, the isoaspartyl dipeptidase responsible for the final step of CGP catabolism in vegetative cells (ISO) was significantly reduced across all four overexpression lines. ISO was reduced by 50% with P*_petE_*::*gifA* in the WT background (Fig. 6e) and 80% in both overexpression lines in the Δ*amt* background (Fig. 6f). The simultaneous accumulation of CGP, induction of the enzyme which catalyzes its synthesis, and suppression of its catabolism across all IF7A overexpression lines suggest that the inhibition of heterocyst GS activity influences the global N economy of the filament by shifting metabolism away from Gln-based assimilation and distribution, toward the storage of CGP.

### MSX-mediated GS inhibition directly demonstrates the role of Amt transporters in nitrogen recovery

The consistent amplification of all phenotypes in the Δ*amt* genetic background suggests that Amt transporters play a role in recycling N lost by passive diffusion under diazotrophic conditions. Under strictly diazotrophic conditions, a fraction of the NH_3_ produced by nitrogenase that escapes GS-mediated assimilation can diffuse passively out of the filament down the concentration gradient. Upon reaching the external medium at pH 8.0, this NH_3_ is rapidly protonated to NH_4_^+^, generating a pool of charged ammonium that cannot reenter the cell by passive diffusion and therefore requires active transport for recovery. Upon diazotrophic conditions, we hypothesize that Amt transporters serve this function and that their absence in the Δ*amt* background results in a chronic drain on the internal N pool that amplifies the consequences of GS inhibition. However, we were unable to detect any excreted ammonium in the culture medium with any of the strains grown under diazotrophic conditions. This suggests that under physiological conditions, the rate of passive NH_3_ diffusion was low, and the resulting extracellular NH_4_^+^ accumulation fell below the detection limit of the colorimetric assay.

To overcome this limitation, we inhibited GS irreversibly with 3 µM MSX. This is expected to increase the intracellular NH_3_ pool available for diffusion by blocking its assimilation, thereby amplifying N leakage to improve the detectable levels. The WT, Δ*amt*, and the corresponding P*_petE_*::*gifA* overexpression lines, which showed the greatest reduction of GS activity in this study, were all treated with MSX, and the ammonium accumulation in the culture medium was monitored over 96 h (Fig. 7).

**Figure 7:**
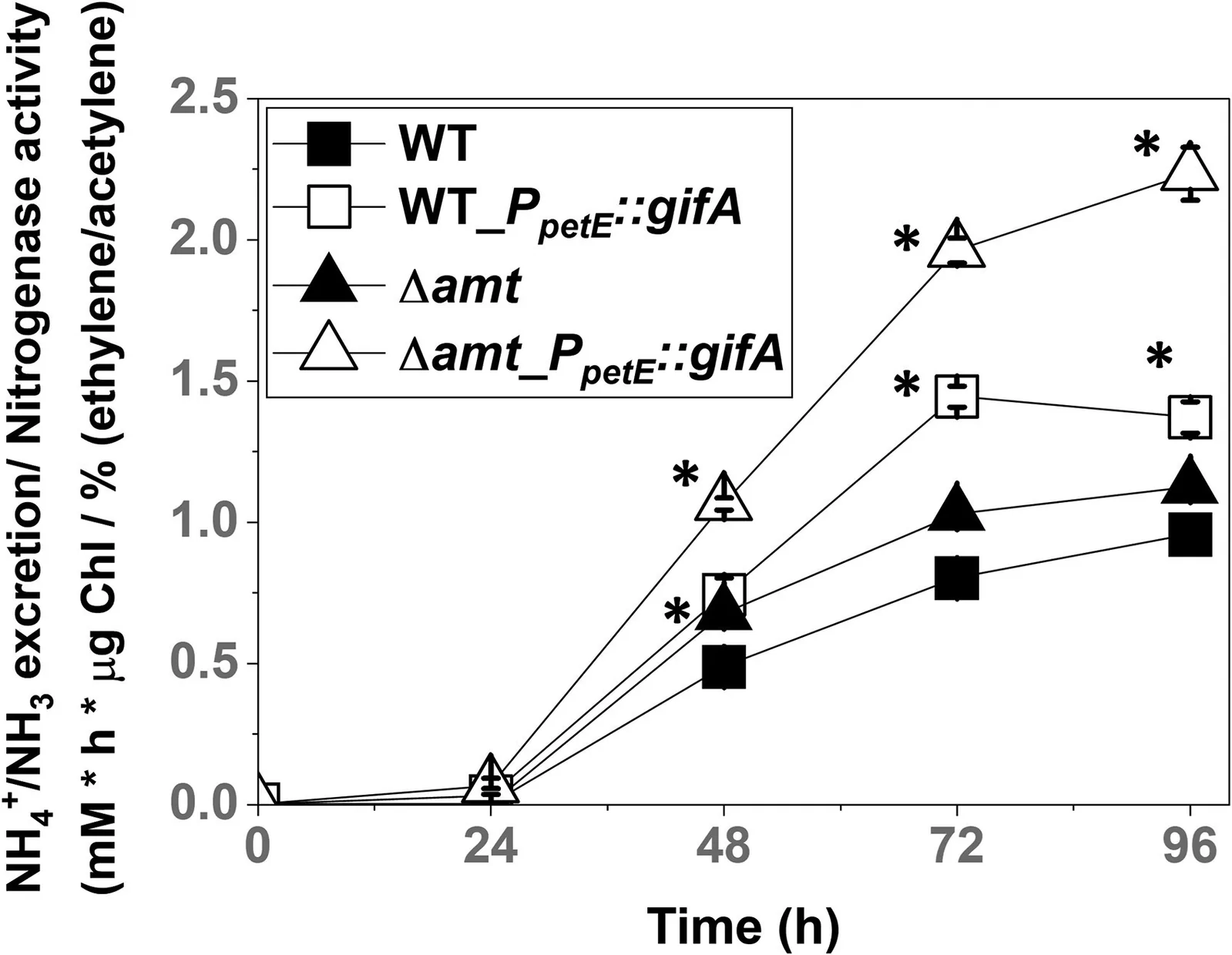
Ammonium excretion assay. Ammonium/ammonia accumulation in the culture medium (NH_4_^+^/NH_3_ excretion, normalized over the nitrogenase activity) over 96 h for WT (filled squares), WT_P*_petE_::gifA* (open squares), Δ*amt* (filled triangles), and Δ*amt*_P*_petE_::gifA* (open triangles) strains. All strains were treated with 3 µM MSX from t = 0 h under diazotrophic growth conditions. Ammonium excretion was undetectable in all strains prior to MSX addition. Data are expressed as mean ± SD of at least three biological replicates. Asterisks indicate statistically significant differences between genetic backgrounds (Student's t-test, *P*-value < 0.05).

Upon MSX treatment, ammonium excretion was detected in all four strains, suggesting that GS is the primary route of NH_3_ assimilation in *Anabaena* and that its inhibition results in the release of fixed N into the medium.

The Δ*amt* strain excreted significantly more ammonium than WT under MSX treatment throughout the time course. When the MSX treatment was applied to strains carrying also the genetic inhibition of GS (P*_petE_*::*gifA*), the difference between the two genetic backgrounds was amplified (Fig. 7), demonstrating that Amt transporters are involved in the recovery of the fraction of lost N by passive diffusion out of the filament. Consequently, the absence of Amt transporters likely explains why all the metabolic phenotypes observed in the Δ*amt* background throughout this study were amplified.

## Discussion

The posttranslational regulation of GS by small inhibitory factors represents a conserved and rapid mechanism for adjusting N assimilation capacity in cyanobacteria (García-Domínguez et al. 1999; Galmozzi et al. 2010). In *Anabaena*, this regulatory layer operates within a multicellular context in which vegetative cells and heterocysts must coordinate their N economies. By driving IF7A overexpression specifically in heterocysts (P*_nifHDK_*::*gifA*) or in the whole filament (P*_petE_*::*gifA*), this study provides the first cell-type-resolved dissection of GS function in filamentous diazotrophic cyanobacteria like *Anabaena*. The manipulation of IF7A levels provides a uniquely precise way for perturbing GS activity without altering the structural genes of the GS/GOGAT cycle itself, therefore preserving the integrity of the transcriptional regulatory network (eg NtcA, PII, and PipX) and allowing its downstream responses to be interpreted as genuine consequences of altered N assimilation flux rather than artifacts of changes in gene expression. It is also worth noting that the 2-/3-fold increase in IF7A abundance achieved in the overexpression lines (Fig. 2a and b) falls within the range of variation that occurs in vivo in response to changes in the N_2_ status (Galmozzi et al. 2010; Perin et al. 2021), suggesting that the phenotypes described in this work are physiologically relevant responses.

### GS in vegetative cells is rate-limiting for the growth of the filament upon diazotrophic conditions

Whereas the heterocyst-targeted GS inhibition produced a moderate growth reduction, the whole-filament inhibition caused near-complete growth arrest in both genetic backgrounds (Fig. 2c and d). This impact on growth was paralleled by a similar trend on photosynthetic performance, as the Chl content and Φ_PSII_ were severely reduced in P*_petE_*::*gifA* strains regardless of the genetic background (Table 1). These results suggest that GS in vegetative cells is the primary rate-limiting step for filament growth under N_2_ fixation conditions. Vegetative cells constitute most of the cellular mass of the filament and are responsible for the bulk of C fixation (Santin et al. 2026) and biosynthetic activity, making their ability to reassimilate ammonium derived from catabolic reactions, critical for the overall N balance of the filament. When vegetative cell GS inhibition is sustained over time, we observed the onset of its proteolytic degradation in the whole filament (Figure S2a), likely to recycle assimilable N, which is consistent with the GS protein turnover upon prolonged inactivation reported in *Synechocystis* (Galmozzi et al. 2010).

The severe reduction of the Chl content and apparent Φ_PSII_ in the P*_petE_*::*gifA* strains, regardless of the genetic background, are consistent with a N starvation-like response, in which Chl biosynthesis and photosystem assembly are impaired due to shortage of Gln as a N donor for tetrapyrrole and phycobilisome biosynthesis (Collier and Grossman 1994).

### GS in heterocysts regulates the routing of fixed nitrogen

The cell-type-specific localization of the proteins quantified in this work provides a key interpretative framework for understanding the systemic consequences of the cell-type-specific GS inhibition. For proteins present in both cell types (eg GS, NtcA, PII, and PipX), the whole-filament nature of our measurements does not allow to resolve the relative contributions of the two cell types to the observed responses. For cell-type-specific proteins (eg CphA1 and nitrogenase, which are heterocyst-localized, or ISO and GOGAT which are specific of vegetative cells), we can distinguish between two types of response: cell-autonomous responses, in which a protein abundance/activity changes in the same cell type where GS is inhibited, and systemic responses, in which a protein changes in the other cell type with respect to the site of GS inhibition. Proteins that respond with similar magnitude in P*_nifHD_K*::*gifA* and P*_petE_*::*gifA* strains demonstrate that GS activity in heterocysts is sufficient to regulate their abundance/activity.

As described in the Results, nitrogenase activity and content, CphA1 abundance and CGP accumulation, and ISO suppression all responded with similar magnitude in all IF7A overexpression strains (Figs. 4 and 6), indicating that GS activity in heterocysts regulates both the rate of N_2_ fixation itself and the metabolic direction of freshly fixed N. When heterocyst GS is active, NH_3_ is efficiently incorporated into Gln and exported to vegetative cells, and nitrogenase activity is sustained. When it is inhibited, fixed N is diverted into CGP storage, its catabolism in vegetative cells is simultaneously suppressed, and nitrogenase activity is downregulated, consistent with the feedback downregulation of the NtcA–PII–PipX signaling axis (Fig. 5) that attenuates the transcriptional activation of N_2_ fixation genes, likely because of the reduced Gln availability. It is also worth noting that GOGAT abundance responded to the heterocyst GS inhibition in both genetic backgrounds (Figure S2), strengthening the conclusion that heterocyst GS activity is required for routing fixed N toward the downstream enzymatic machinery of N assimilation across the whole filament.

A comparison of the GS activity reductions in *P_nifHDK_*::*gifA* and P*_petE_*::*gifA* in the Δ*amt* background allows an indirect estimation of the relative contributions of the two cell types to the whole-filament GS activity. The heterocyst-specific inhibition reduced GS activity from ∼0.095 to ∼0.045 ΔOD_850_, while the additional inhibition in vegetative cells in P*_petE_*::*gifA* further reduced activity to ∼0.012 ΔOD_850_ (Fig. 3). This suggests that heterocysts account for approximately 50% of the whole-filament GS activity, despite representing on average only ∼10% of the total cell number (Santin et al. 2026). This implies a substantially higher GS specific activity per cell in heterocysts relative to vegetative cells, consistent with their primary role in the rapid assimilation of NH_3_ produced by nitrogenase and with the differential expression of GS between the two cell types previously reported at the transcript level (Galmozzi et al. 2010).

Overall, our results reveal a fundamental division of labor in which GS in vegetative cells primarily sustains growth, likely from the reassimilation of ammonium derived from catabolic reactions, while GS in heterocysts simultaneously determines the efficiency of N_2_ fixation, the routing of fixed N between distribution and storage, and the capacity of vegetative cells to recycle CGP-derived N compounds (Fig. 8). A comparable asymmetry between the roles in N assimilation of the two cell types has been inferred indirectly from heterocyst differentiation mutants in the past (Martín-Figueroa et al. 2000; Herrero et al. 2004; Flores et al. 2019b), but our results provide direct experimental evidence using a gain-of-function approach that leaves filament organization intact.

**Figure 8:**
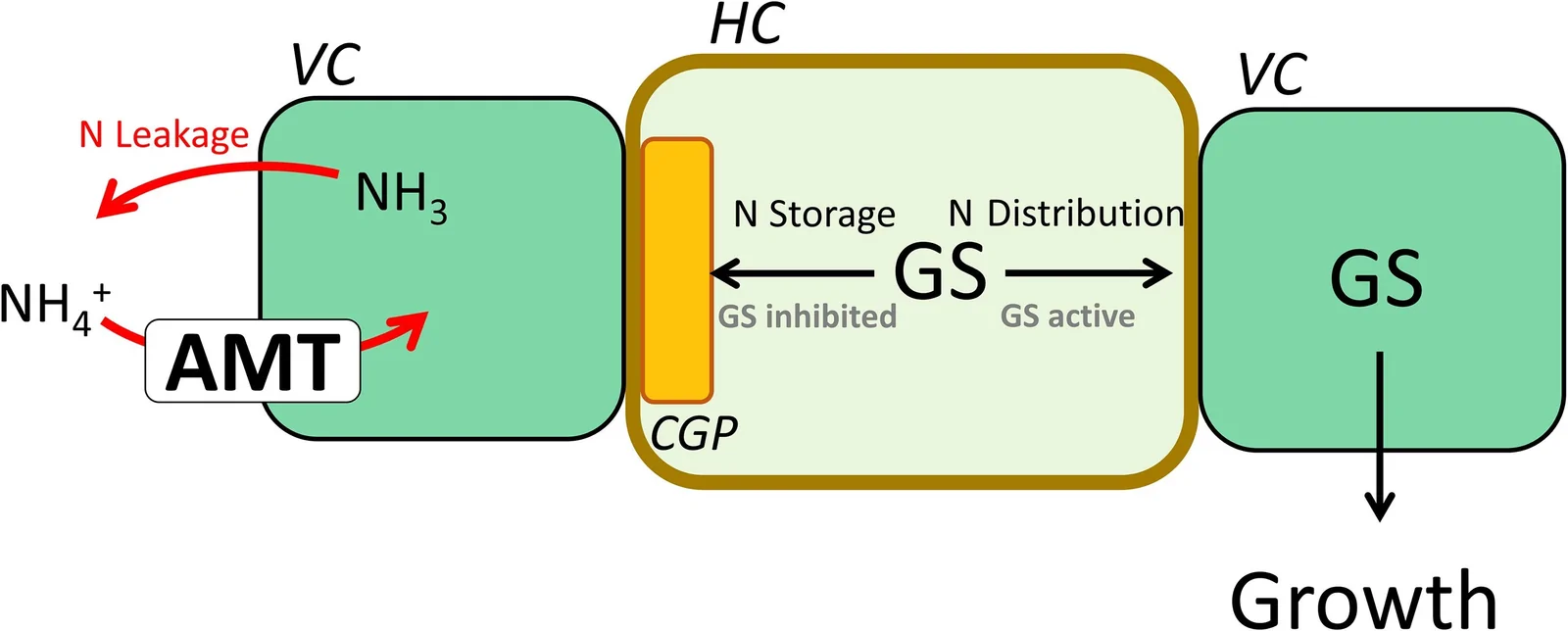
Schematic model of the cell-type-specific roles of glutamine synthetase and Amt transporters in the nitrogen economy of the diazotrophic filament of *Anabaena* sp. PCC 7120. In vegetative cells (VC), GS activity is rate-limiting for filament growth, sustaining biosynthesis likely from the reassimilation of ammonium derived from catabolic reactions (right). In heterocysts (HC), GS regulates the routing of freshly fixed nitrogen between two alternative fates: when active, it channels NH_3_ for export to vegetative cells (N distribution); when inhibited, fixed nitrogen is diverted into cyanophycin granule polypeptide (CGP), while its catabolism in vegetative cells is simultaneously suppressed (N storage). The physiological transition between these two states is regulated by the C–N balance of the filament through the NtcA–PipX–PII signaling cascade, which modulates GS posttranslational activity in response to the nitrogen status of the cell. NH_3_ produced by nitrogenase that escapes GS-mediated assimilation diffuses passively out of the filament (N leakage), where it is protonated to NH_4_^+^ and actively recovered by Amt transporters (AMT, red arrows), recycling fixed N that would otherwise be permanently lost. VC, vegetative cell; HC, heterocyst; AMT, ammonium transporter; GS, glutamine synthetase; CGP, cyanophycin granule polypeptide; N, nitrogen.

When GS activity in heterocysts is inhibited, the simultaneous accumulation of CGP, suppression of its catabolism, and reduction of nitrogenase activity collectively induce a self-reinforcing N sequestration loop that progressively depletes the bioavailable N pool and drives the reduced growth phenotype observed in P*_nifHDK_*::*gifA* overexpression strains. A similar functional framework, in which N storage competes with distribution when N assimilation is impaired, has also been proposed in unicellular cyanobacteria (Forchhammer and Selim 2020), suggesting that the functional coupling between N assimilation and storage represents a conserved N homeostasis strategy across cyanobacteria. The majority of ammonium produced by nitrogenase in the heterocyst is incorporated into C-skeletons by the GS–GOGAT pathway (Wolk et al. 1976). With an IF7A-inhibited GS, ammonium may accumulate to a level that allows glutamate dehydrogenase to produce glutamate from 2-OG, as has been shown for MSX-treated cells (Meeks et al. 1977). Glutamate can then be a source of arginine and aspartate for CGP biosynthesis (Flores et al. 2019a).

The findings reported here are particularly timely in the context of the recent characterization of the nitroplast, a N_2_-fixing organelle evolved from a cyanobacterial endosymbiont in the marine alga *Braarudosphaera bigelowii* (Coale et al. 2024). Like the heterocyst–vegetative cell interface in *Anabaena*, the nitroplast–host interface represents a metabolic junction at which fixed N must be efficiently transferred from the fixing compartment to the metabolically active recipient. Our data highlight that this transfer is likely governed by GS activity in the fixing compartment and that its impairment diverts fixed N into storage rather than distribution, with implications also for any N_2_-fixing symbiosis or organelle. The mechanistic framework established here may therefore inform the design of synthetic N_2_-fixing systems and the interpretation of metabolic integration in naturally evolved diazotrophic associations.

### IF7A overexpression propagates to the NtcA–PII–PipX signaling axis

IF7A overexpression consistently reduced PipX abundance regardless of cell-type selectivity or genetic background (Fig. 5), connecting posttranslational GS inhibition to a systemic attenuation of the N starvation transcriptional response. Since PipX is required as a coactivator for NtcA-dependent gene activation, including N_2_ fixation genes, its reduction provides a mechanistic basis for the concurrent downregulation of the nitrogenase complex (Fig. 4) (Espinosa et al. 2006; Llácer et al. 2010; Valladares et al. 2011).

This contrasts with the classical model of NtcA–PII–PipX signaling, which predicts that GS inhibition, by reducing Gln synthesis, would increase the 2-OG/Gln ratio and thereby activate NtcA and its downstream targets. However, it is worth noting that the current mechanistic understanding of this signaling network is largely built on transcriptional data collected over short time windows, typically the first few hours following N step-down (Valladares et al. 2011; Forchhammer and Selim 2020), while the long-term protein-level consequences of sustained N limitation remain poorly characterized. Instead, the data in this manuscript quantify NtcA, PII, and PipX protein abundance under chronic diazotrophic conditions after 96 h. Under these conditions, the depletion of the Gln pool reaches a severity (Perin et al. 2021) sufficient to globally compromise protein synthesis and stability, impairing the accumulation of regulatory proteins including NtcA and PipX themselves, an effect that would not be captured by short-term transcriptional studies. Alternatively, reduced PipX availability may reflect a redistribution toward its alternative binding partner PipY/SbtB, whose interaction with PipX is modulated by nucleotide and metabolite signals integrating the C–N status (Forcada-Nadal et al. 2018; Forchhammer and Selim 2020).

It is also worth noting that the observed alterations in the GS/GOGAT cycle and NtcA–PII–PipX signaling axis reflect specific N assimilation remodeling rather than generalized metabolic perturbations. Isocitrate dehydrogenase (IDH) catalyzes the conversion of isocitrate to 2-OG in the TCA cycle and is the primary source of 2-OG in *Anabaena*, the metabolite that serves as both the C skeleton for Glu biosynthesis via GOGAT and the key signaling molecule of the NtcA–PII–PipX cascade (Muro-Pastor et al. 2001; Zhang et al. 2018). The stability of IDH across all overexpression strains and genetic backgrounds (Figure S4a and b) indicates that the changes described in this work are therefore not driven by an upstream shortage of the 2-OG substrate.

### Amt transporters sustain internal nitrogen cycling by recovering leaked ammonia

All phenotypes described in this work were amplified in the Δ*amt* genetic background (Figs. 2–6), and the inhibitory effect of IF7A on GS activity, absent in the WT (Fig. 3a and c), became fully apparent only when Amt transporters were absent (Fig. 3b and d), highlighting that the internal N economy is more vulnerable to GS inhibition when Amt-mediated N recycling is compromised. The saturation kinetics profile of the GS abundance–activity relationship in the Δ*amt* background (Fig. 3f) provides additional mechanistic insight into the nature of the IF7A-mediated GS inhibition. This nonlinear relationship indicates that, at low GS abundance, activity drops disproportionately, consistent with IF7A acting as a stoichiometric inhibitor that, when present in excess relative to GS, occupies an increasing fraction of the available enzyme molecules and drives a reduction of their specific activity. When GS abundance is high, IF7A is sub-stoichiometric relative to GS, and a sufficient fraction of enzyme molecules remains uninhibited to sustain measurable activity. However, as GS abundance decreases in the overexpression lines, the IF7A/GS ratio increases, and the inhibited fraction grows nonlinearly, producing the observed steep drop-in activity at low GS abundance. This behavior implies the existence of a critical threshold of GS abundance below which IF7A-mediated inhibition becomes essentially complete, a threshold that the Δ*amt* overexpression strains fall below but that the WT overexpression strains do not reach, likely explaining the genetic background-dependent unmasking of the IF7A–GS-inhibitory relationship.

This genetic background-dependent unmasking of the IF7A–GS-inhibitory relationship indicates that Amt transporters likely compensate the inhibition of GS in the WT. Because all experiments in this study were conducted under strictly diazotrophic conditions, with atmospheric N_2_ as the sole N source, this function of Amt transporters cannot be attributed to their external ammonium uptake activity.

Upon diazotrophic conditions, the NH_3_ produced by nitrogenase that escapes GS-mediated assimilation can diffuse passively out of the filament as uncharged NH_3_ down its concentration gradient. Upon reaching the external medium, this NH_3_ is rapidly protonated to NH_4_^+^ (at pH 8.0), generating a pool of charged ammonium that cannot reenter the cell by passive diffusion and therefore requires active transport for recovery. Upon diazotrophic conditions, we propose that Amt transporters serve precisely this function in diazotrophic cyanobacteria, by recycling fixed N that would otherwise be permanently lost. In the Δ*amt* background, this reabsorption capacity is absent, resulting in a more severe internal N limitation that amplifies the consequences of GS inhibition. Under physiological conditions, we could not detect any excreted ammonium in the culture medium, likely because the basal rate of passive NH_3_ diffusion falls below the detection limit of standard colorimetric assays. MSX treatment overcomes this limitation by irreversibly inhibiting GS and thereby increasing the intracellular NH_3_ pool available for diffusion. The significantly greater ammonium excretion of the Δ*amt* strain with respect to the WT under MSX treatment (Fig. 7) and its further amplification in Δ*amt*_P*_petE_*::*gifA* with respect to WT_P*_petE_*::*gifA* directly demonstrate that Amt transporters recover extracellular NH_4_^+^ that accumulates following passive NH_3_ leakage (Fig. 8) and that genetic GS inhibition and Amt deletion act additively on the same N recycling process. This recycling of ammonium has also been shown for unicellular cyanobacteria such as *Synechococcus* sp. PCC 7942 (Vázquez-Bermúdez et al. 2002a).

Together, these observations are consistent with the lower basal level of IF7A in the Δ*amt* background with respect to the WT (Fig. 2b), highlighting a filament operating under chronic relative N limitation, as previously observed (Perin et al. 2021), and therefore maintaining GS in a more active state (Fig. 3b).

Such Amt-mediated N recycling is expected to be particularly relevant in nature where diazotrophic cyanobacteria typically inhabit N-limited environments where they fix atmospheric N_2_ as a primary N source. In these conditions, any fixed N lost by passive diffusion represents an irreversible metabolic cost that cannot be offset by external uptake, making Amt-mediated recycling an essential determinant of the efficiency of biological N_2_ fixation (Muro-Pastor et al. 2005; Galmozzi et al. 2010).

## Conclusions

This study reveals a fundamental division of labor between the GS in the two cell types of diazotrophic filamentous cyanobacteria like *Anabaena*. Glutamine synthetase in vegetative cells is the rate-limiting node for the diazotrophic growth, while GS in heterocysts governs the routing of freshly fixed N, either toward distribution or cyanophycin storage. Amt transporters play an essential role in the nitrogen economy of the diazotrophic filament by actively recovering NH_4_^+^ that accumulates extracellularly following passive NH_3_ diffusion, a constitutive nitrogen recycling function that prevents chronic nitrogen loss and is expected to buffer the metabolic consequences of fluctuations in GS activity, likely in nature. Together, these findings redefine the architecture of intercell-type nitrogen homeostasis in diazotrophic filamentous cyanobacteria and establish cell-type-specific GS function as a central determinant of how nitrogen is balanced toward distribution and storage in nitrogen-limited environments. As the field moves toward understanding and engineering nitrogen-fixing associations, the molecular principles governing nitrogen routing at the interface between fixing and assimilating compartments described here will provide an essential mechanistic foundation.

## Supplementary Material

kiag745_Supplementary_Data

## Data Availability

The data underlying this article are available in the article and in its online supplementary material.
