# Supplementary material for: Cell-type-specific glutamine synthetase inhibition reveals the intercellular nitrogen economy in Anabaena sp. PCC 7120
Source: Plant Physiol. 2026 Oct 6;202(2):kiag745. doi: 10.1093/plphys/kiag745 (PMC13673380; doi:10.1093/plphys/kiag745)
Supplement: Supplementary file 1 [file kiag745_supplementary_data.pdf]

Supporting Information

Cell-type-specific glutamine synthetase inhibition reveals the intercellular nitrogen economy in *Anabaena* sp. PCC 7120

David Malatinszky[1], Mattia Storti[2], Enrique Flores[3], Patrik Jones[1] and Giorgio Perin[1,2]*

1 Department of Life Sciences, Imperial College London, London, UK
2 Department of Biology, University of Padova, 35131, Padova, Italy
3 Instituto de Bioquímica Vegetal y Fotosíntesis, CSIC and University of Seville, Seville, Spain

*Corresponding author: Giorgio Perin. Department of Biology, University of Padova, 35131, Padova, Italy. Email: giorgio.perin@unipd.it

The author responsible for distribution of materials integral to the findings presented in this article in accordance with the policy described in the Instructions for Authors (https://academic.oup.com/plphys/pages/General-Instructions) is Giorgio Perin

Running head: GS and Amt control N homeostasis in *Anabaena*

Supporting Figures

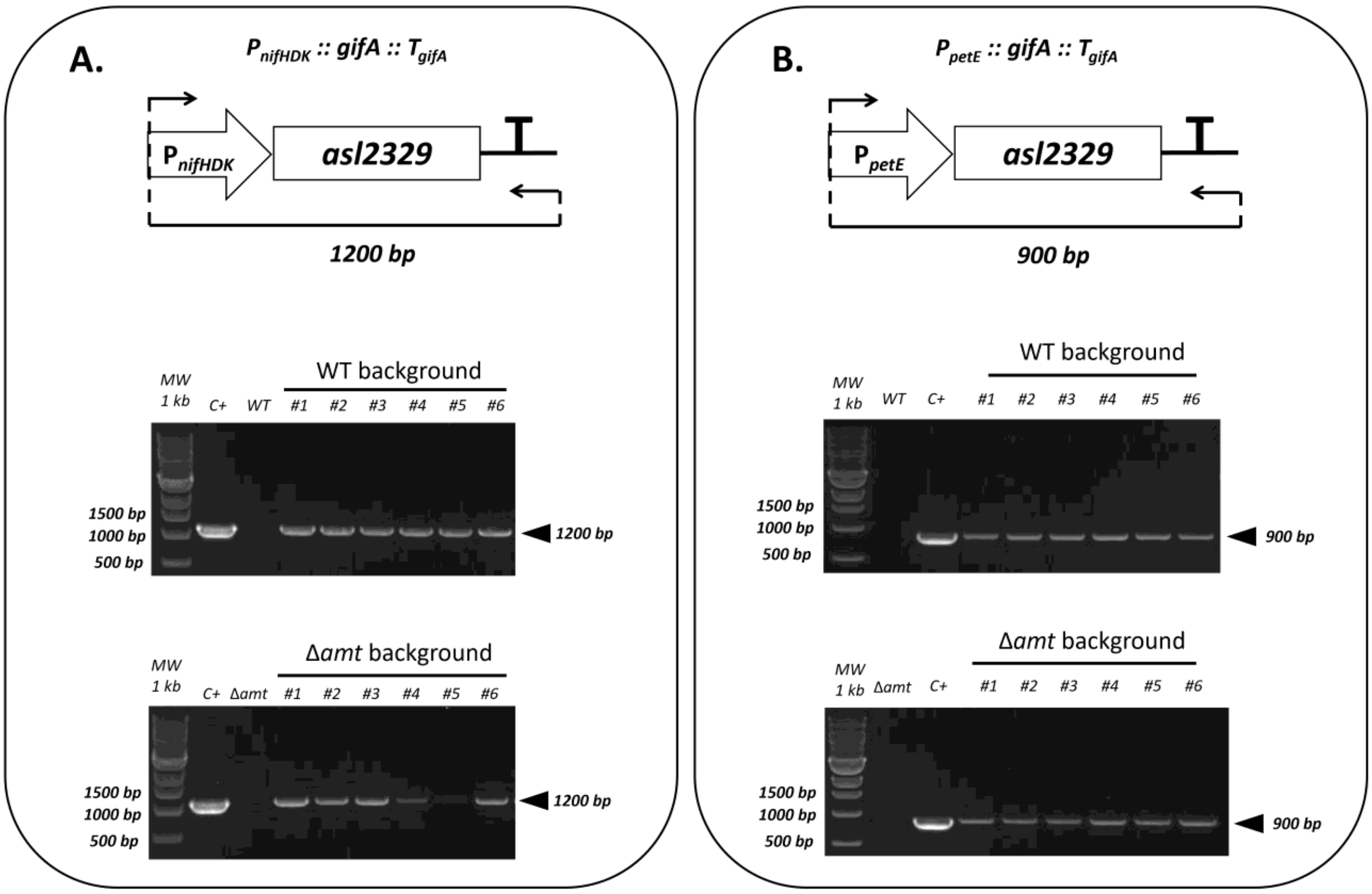

Supplementary Figure S1. Molecular validation of IF7A overexpression lines by colony PCR. *Schematic representation of the two expression constructs (A: P_{nifHDK}::gifA::T_{gifA}, 1,200 bp diagnostic band; B: P_{petE}::gifA::T_{gifA}, 900 bp diagnostic band) and corresponding colony PCR verification gels for independent transformants (#1–#6) in WT (top) and Δamt (bottom) backgrounds. C+: positive control (plasmid DNA); WT or Δamt: parental strain negative controls. All selected transformants produced the expected diagnostic band, confirming correct transformation with the expected IF7A overexpression construct. MW: 1 kb DNA ladder.*

Alt-Text: *Schematic diagrams of two expression constructs followed by agarose gel images of colony PCR verification. Left panel: P_{nifHDK}::gifA construct with expected 1,200 bp diagnostic band confirmed in six independent transformants in both WT and Δamt backgrounds. Right panel: P_{petE}::gifA construct with expected 900 bp diagnostic band confirmed in six independent transformants in both WT and Δamt backgrounds. Parental strains show no band; positive controls show the expected band size.*

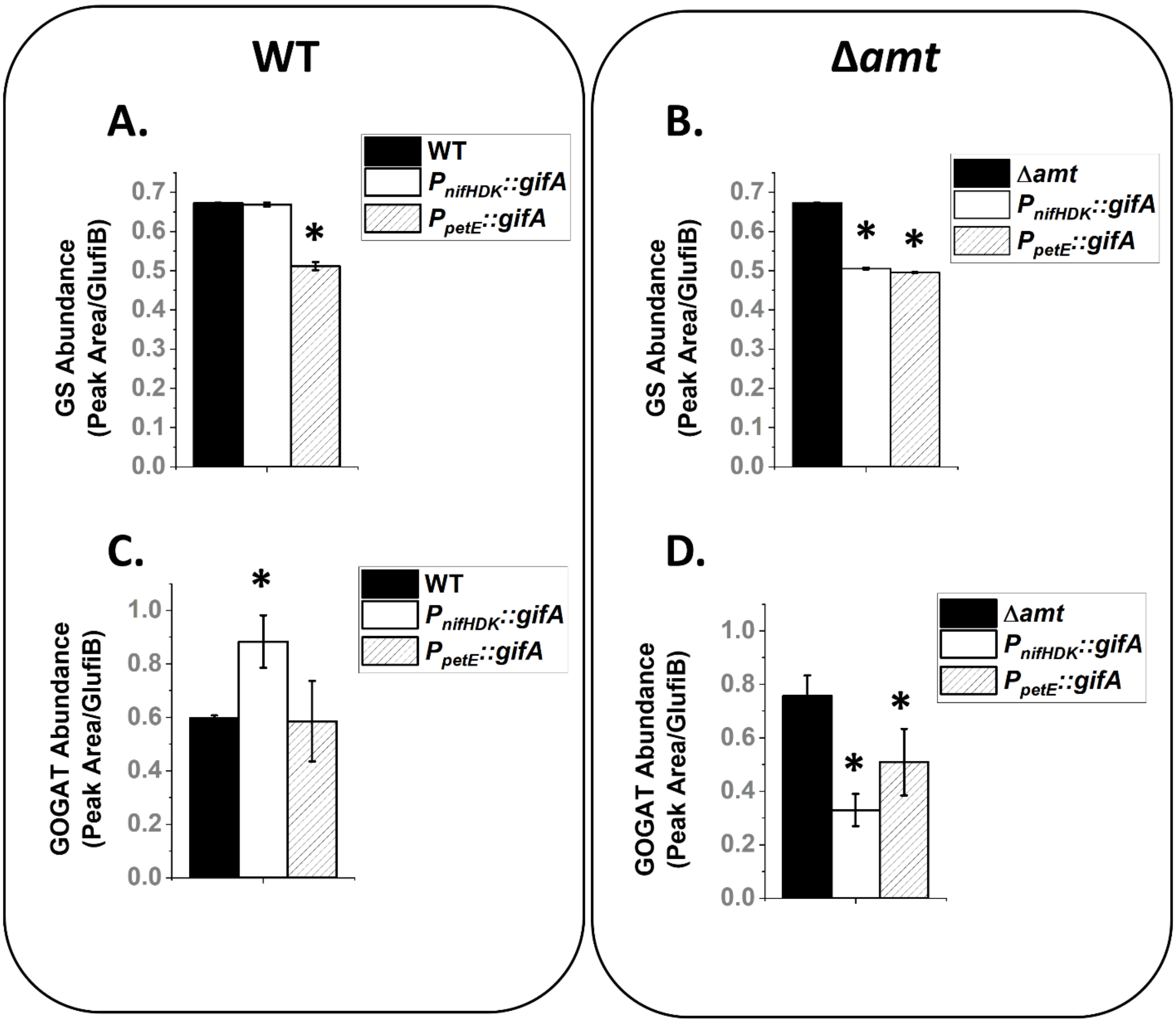

Supplementary Figure S2. Abundance of GS and GOGAT proteins. *(A, B) GS protein abundance (Peak Area/GluFiB) in WT (A) and Δamt (B) parental strains (black bars) and corresponding P_{nifHDK}::gifA (white bars) and P_{petE}::gifA (dashed bars) overexpression lines. (C, D) GOGAT protein abundance (Peak Area/GluFiB) in WT (C) and Δamt (D) backgrounds. Data were collected after 96h of growth in diazotrophic conditions and are expressed as mean ± SD of at least three biological replicates. Protein abundances are normalised to the internal standard GluFiB. Asterisks indicate statistically significant differences between IF7A overexpressing lines and the respective parental strain within the same genetic background (Student's t-Test, p-value < 0.05).*

Alt-Text: *Bar charts showing GS protein abundance unaffected by heterocyst-specific inhibition in both genetic backgrounds but significantly reduced by whole-filament inhibition. GOGAT protein abundance is significantly increased by heterocyst-specific inhibition in the WT background but significantly reduced in both overexpression lines in the Δamt background.*

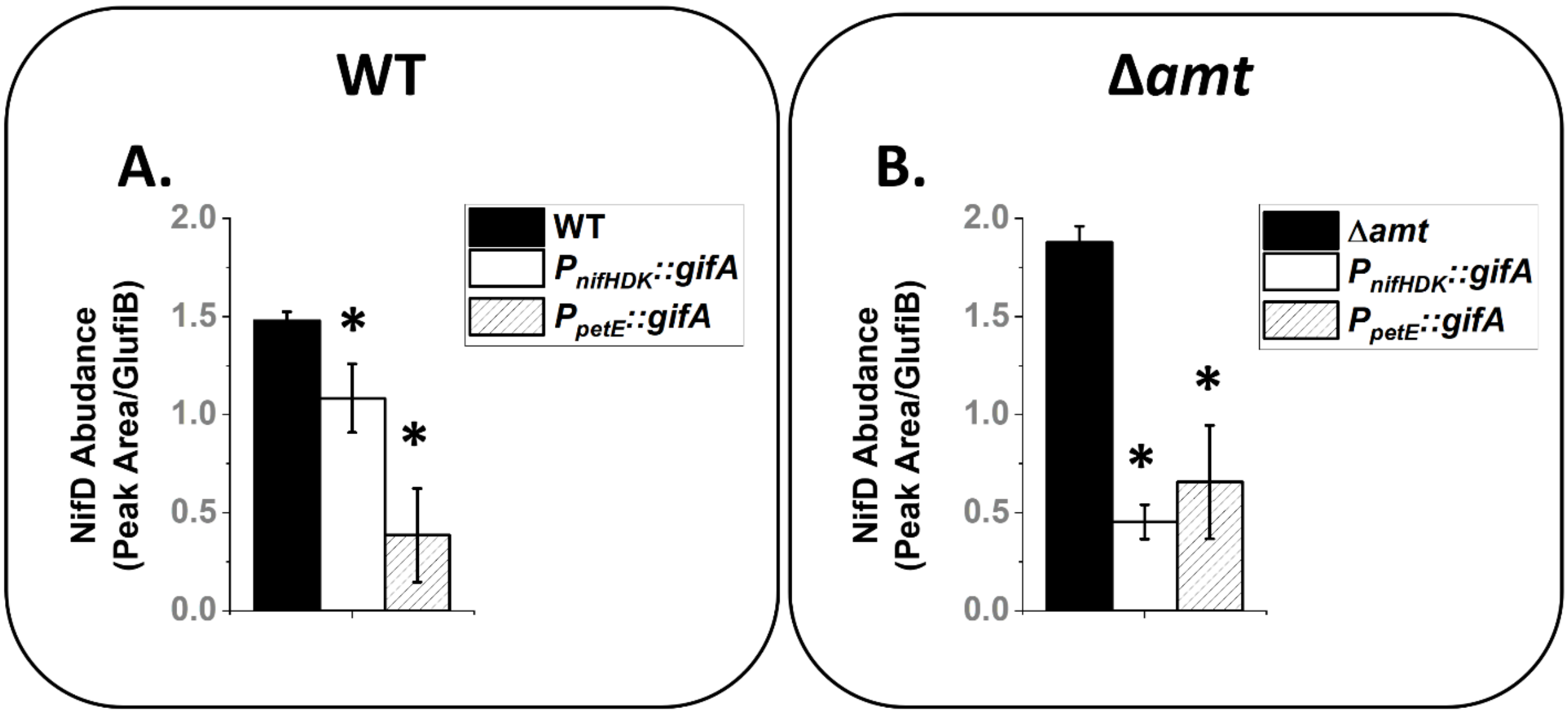

Supplementary Figure S3. Abundance of the nitrogenase α-subunit NifD. *NifD protein abundance (Peak Area/GluFiB) in WT (A) and Δamt (B) parental strains (black bars) and the corresponding* P_{nifHDK}*::gifA (white bars) and* P_{petE}*::gifA (dashed bars) overexpression lines. Protein abundance is normalised to the internal standard GluFiB. Asterisks indicate statistically significant differences between IF7A overexpressing lines and the respective parental strain within the same genetic background (Student's t-Test, p-value < 0.05).*

Alt-Text: *Bar charts showing NifD protein abundance significantly reduced in both overexpression lines relative to parental strains in both WT and Δamt backgrounds.*

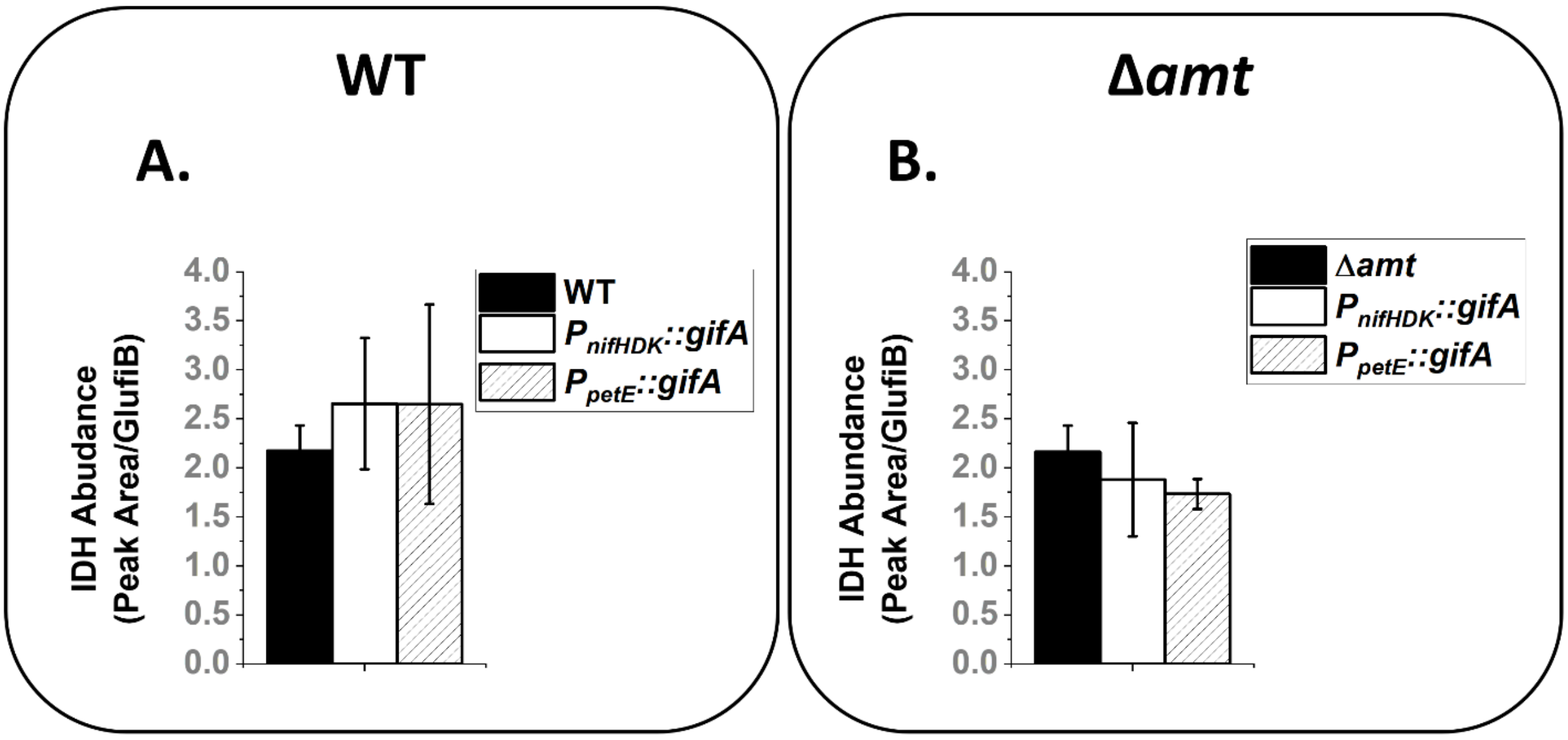

Supplementary Figure S4. Abundance of IDH. *(A, B) Isocitrate dehydrogenase (IDH) protein abundance (Peak Area/GluFiB) in WT (A) and Δamt (B) parental strains (black bars) and the corresponding* P_{nifHDK}*::gifA (white bars) and* P_{petE}*::gifA (dashed bars) overexpression lines. Protein abundance is normalised to the internal standard GluFiB.*

Alt-Text: *Bar charts showing IDH protein abundance unchanged across all overexpression lines in both WT and Δamt backgrounds.*

Supporting Tables

Supplementary Table S1. Primer sequences for cloning. *Sequences are written 5' to 3'. Overlapping sequences are in lowercase.*

ID	Name	Sequence
1	RSF1010-bb-F	CTGAAAGCGACCAGGTGC
2	RSF1010-bb-R	GCAGGAGCAGAAGAGCATAC
3	Sm^R-T_{gifA}-F	caggtgttaaTCATGGCTTCTTGTTATGAC
4	Sm^R-RSF1010-R	gagcacctggtcgctttcagATTCTCACCAATAAAAAACGC
5	P_{petE}-RSF1010-F	gtatgctcttctgctcctgcCAGTACTCAGAATTTTTTGCTGAGG
6	P_{petE}-*gifA*-R	gaatagacatGCGTTCTCCTAACCTGTAG
7	*gifA*-P_{petE}-F	aggagaacgcATGTCTATTCAAGAAAAATCTCG
8	*gifA*-R	gaagccatgaTTAACACCTGTTAGCAATTTATAG
9	P_{nifHDK}-RSF1010-F	gtatgctcttctgctcctgcGTTGCGGTTCCTGTTTAGC
10	P_{nifHDK}-gifA-R	gaatagacatTGTTCTCTTTTCCTGCAATTG
11	*gifA*-P_{nifHDK}-F	aaagagaacaATGTCTATTCAAGAAAAATCTCG
12	RSF1010-Psch-seq-F	ATACCATGCTCAGAAAAGGC
13	PSm^R-seq-R	ACATCAAACATCGACCCACG

Supplementary Table S2. Signature peptides for the proteins investigated in this work. *Gene ID, protein ID and corresponding biological function are indicated. Peptide sequences show their position within the corresponding protein in square brackets.*

Gene ID	Protein ID	Biological Function	Peptide Sequences
Alr1827	IDH	Isocitrate dehydrogenase	K.GPLTTPVGGGIR.S [100,111]<br>R.SLNVALR.Q [112,118]<br>K.LDVIVYR.E [146,152]
Alr2328	GS	Glutamine synthetase	K.IELIDLK.F [15,21]<br>K.LGVPIEK.H [205,211]<br>R.IPLSGTNPK.A [348,356]<br>K.NIYELSPEELAK.V [399,410]
Alr4344	GOGAT	Glutamine oxoglutarate aminotransferase	R.FAQVTNPAIDPLR.E [548,560]<br>R.SLSEIIGR.A [1232,1239]<br>K.TLPIVNTDR.T [1310,1318]
Asl2329	IF7A	GS inhibitory factor	SAQELGLPAEELSHYWNPTQGK [29,50]
Alr4392	NtcA	Nitrogen-responsive regulator	K.TIFFPGDPAER.V [32,42]<br>R.ENSVFGVLSLLTGNK.S [70,84]<br>R.LLGDLR.E [192,197]
Ar0485	PipX	PII interacting protein	R.LFFLVGNDIK.A [38,47]<br>K.FQPIGR.T [51,56]
All2319	PII	Nitrogen regulatory protein PII	K.IIAAAR.T [76,81]<br>R.TGEIGDGK.I [82,89]<br>K.IFISPVEQVIR.I [90,100]
All1440	NifK	Nitrogenase MoFe protein β-chain	R.EALTVNPAK.G [59,67]<br>K.AIPEELEIER.G [334,343]<br>R.IGYPLFDR.H [457,464]
All1554	NifD	Nitrogenase MoFe protein α-chain	K.ELIQEVLK.A [14,21]<br>K.LIEELDVLFPLNR.G [135,147]<br>K.IAASLR.E [311,316]
All3879	CphA1	Cyanophycin synthetase	K.AELEQDIQDLK.D [144,154]<br>R.GITIDIR.S [269,275]<br>R.GSASELITK.G [808,816]
All3922	ISO	Isoaspartyl dipeptidase	R.FSGVINVSR.V [93,101]<br>R.GTIGVVALDTYGK.L [178,190]<br>K.LAVGTSTGGK.G [191,200]
Standard	GluFib	[Glu1]-Fibrinopeptide B, human	EGVNDNEEGFFSAR [0,13]
